# Supplementary figures and images for: The molecular mechanism for carbon catabolite repression of the chitin response in Vibrio cholerae
Source: PLoS Genet. 2023 May 12;19(5):e1010767. doi: 10.1371/journal.pgen.1010767 (PMC10208484; doi:10.1371/journal.pgen.1010767)

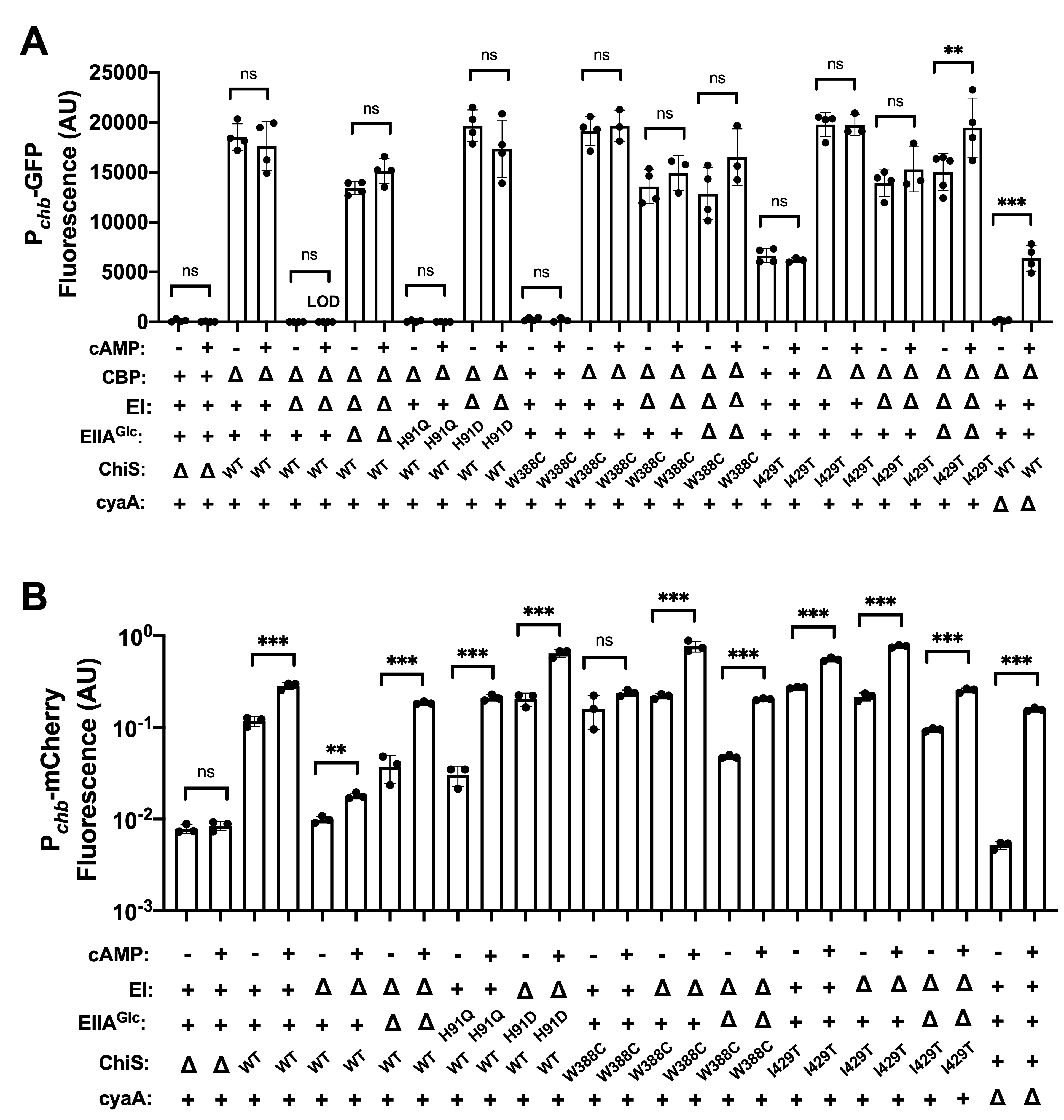

Supplement: S1 Fig — (A) ChiS activation of the chb promoter was assessed in rich medium (in the absence of chitin) using a Pchb-GFP reporter in the indicated strain backgrounds. (B) ChiS activation of Pchb-mCherry was assessed in the indicated strains after cells were incubated on chitin for 48 hrs. mCherry signal was normalized to a constitutively expressed GFP construct. All experiments were performed in the presence or absence of 5 mM exogenous cAMP as indicated. All data for samples where exogenous cAMP was added are identical to that present in Fig 1A and 1B and are included here for ease of comparison. Results are from at least three independent biological replicates and shown as the mean ± SD. Statistical comparisons were made by one-way ANOVA with Tukey’s multiple comparison test. NS, not significant. *** = p < 0.001, ** = p < 0.01, * = p < 0.05. (TIFF) [file pgen.1010767.s001.tiff]

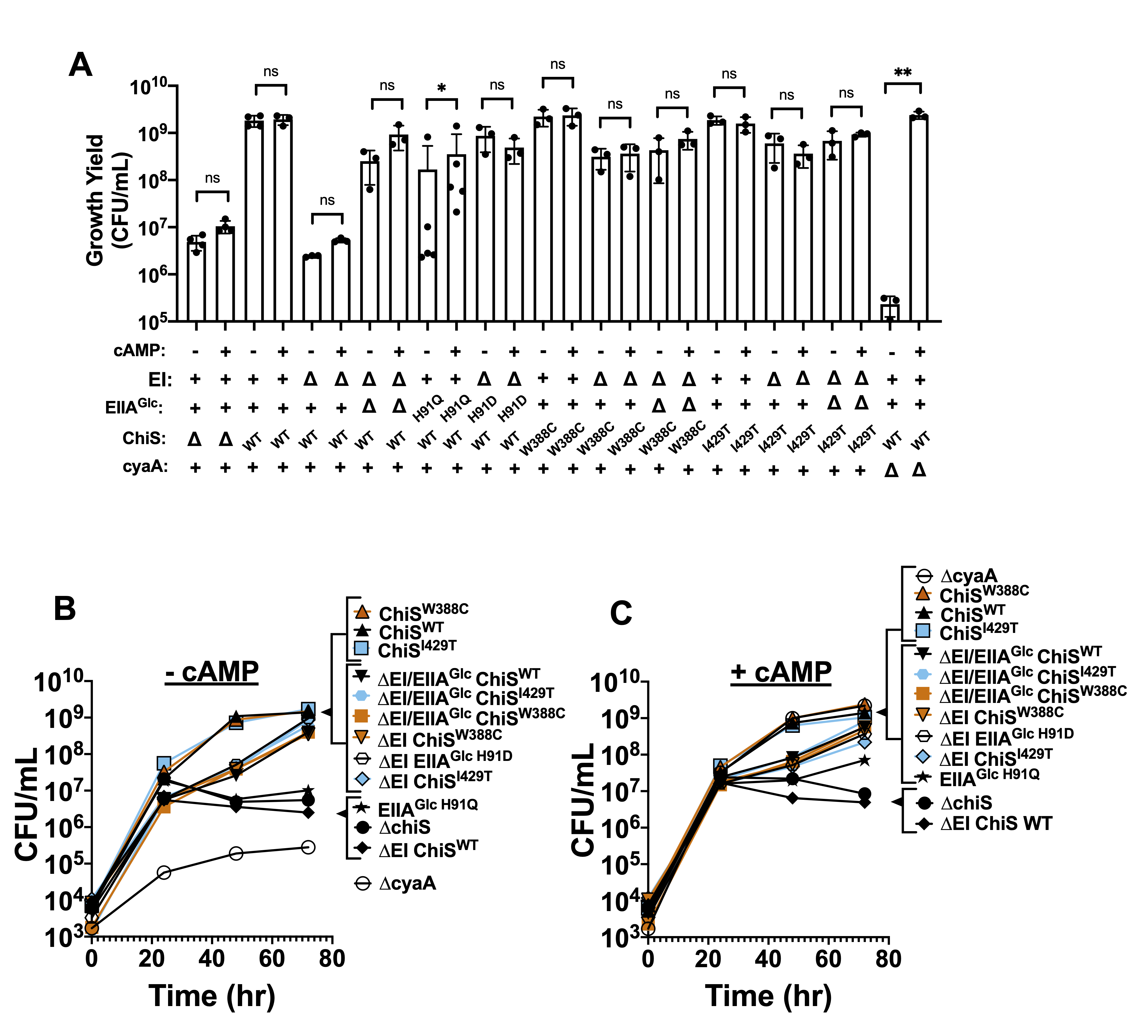

Supplement: S2 Fig — (A) Final growth yield of the indicated strains after a 72 hr incubation in M9 minimal media containing chitin as the sole carbon source. Reactions were supplemented with 5 mM cAMP as indicated. Results are from at least three independent biological replicates and shown as the mean ± SD. Statistical comparisons were made by one-way ANOVA with Tukey’s multiple comparison test. NS, not significant. *** = p < 0.001, ** = p < 0.01, * = p < 0.05. Data for reactions supplemented with cAMP are identical to that presented in Fig 1C and are included here for ease of comparison. (B-C) Representative growth curves of the indicated strains in M9 minimal media containing chitin as the sole carbon source. Reactions either (B) lacked exogenous cAMP or (C) were supplemented with 5mM cAMP. Strains containing suppressor alleles are colored (orange for W388C, blue for I429T). Data are representative of at least three independent biological replicates. (TIFF) [file pgen.1010767.s002.tiff]

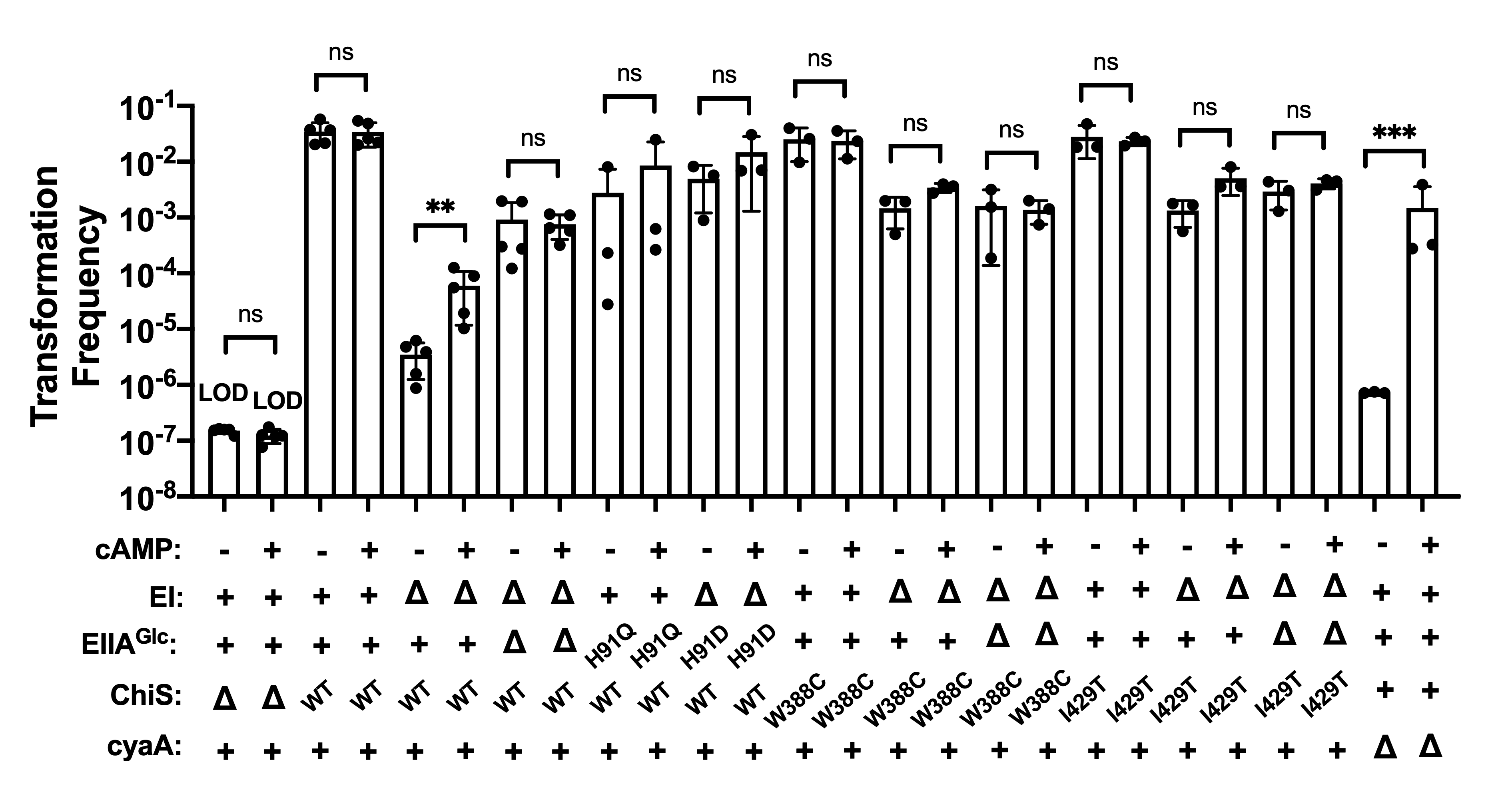

Supplement: S3 Fig — Chitin-induced natural transformation assays of the indicated strains. Reactions were supplemented with 5 mM exogenous cAMP as indicated. All data for samples where exogenous cAMP was added are identical to that presented in Fig 1D and are included here for ease of comparison. Results are from at least three independent biological replicates and shown as the mean ± SD. Statistical comparisons were made by one-way ANOVA with Tukey’s multiple comparison test. NS, not significant. *** = p < 0.001, ** = p < 0.01, * = p < 0.05. LOD, limit of detection. (TIFF) [file pgen.1010767.s003.tiff]

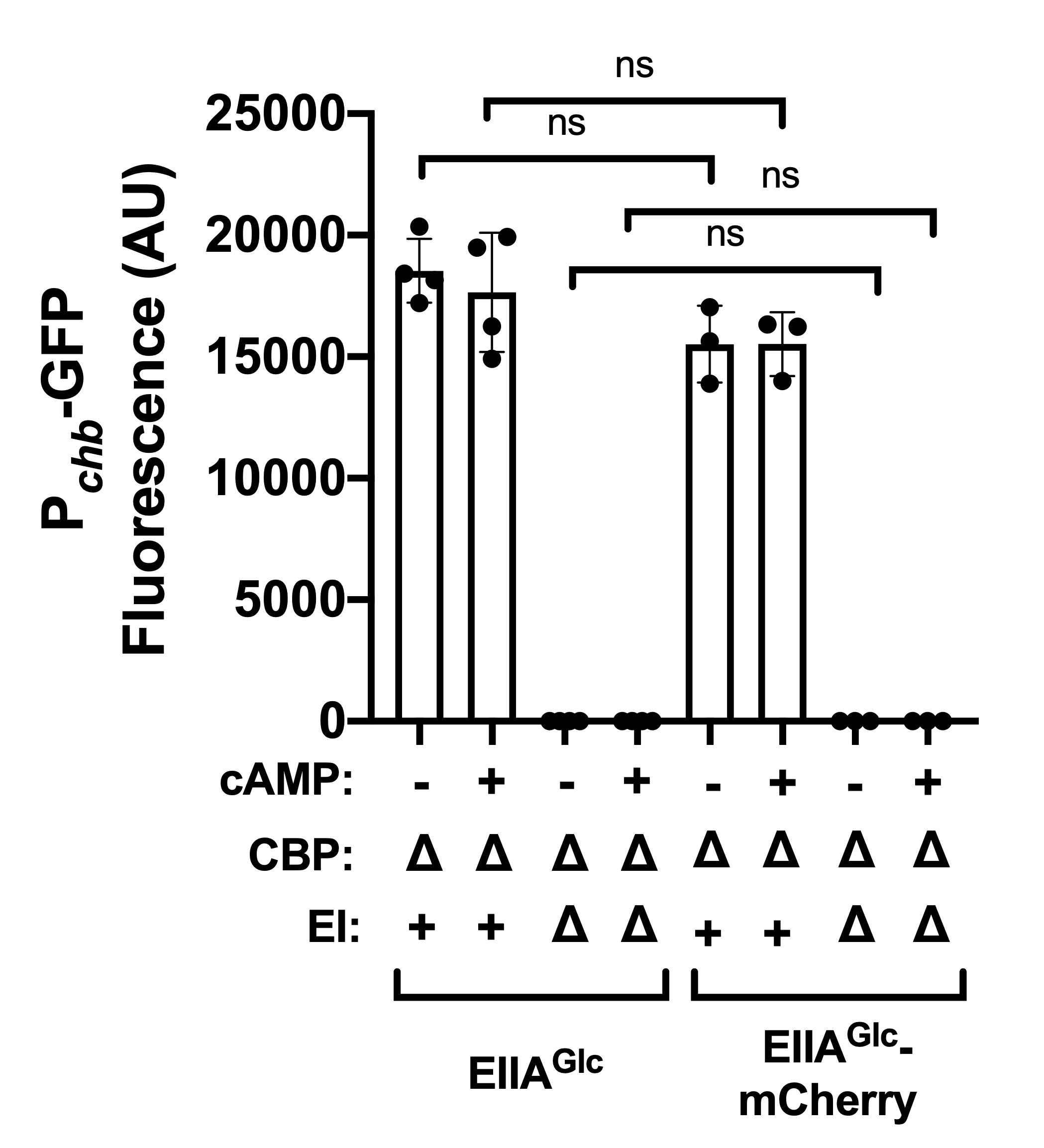

Supplement: S4 Fig — ChiS activation of the chb promoter was assessed in rich medium (in the absence of chitin) using a Pchb-GFP reporter in the indicated strain backgrounds. Results are from three independent biological replicates and shown as the mean ± SD. Statistical comparisons were made by one-way ANOVA with Tukey’s multiple comparison test. NS, not significant. *** = p < 0.001, ** = p < 0.01, * = p < 0.05. (TIFF) [file pgen.1010767.s004.tiff]

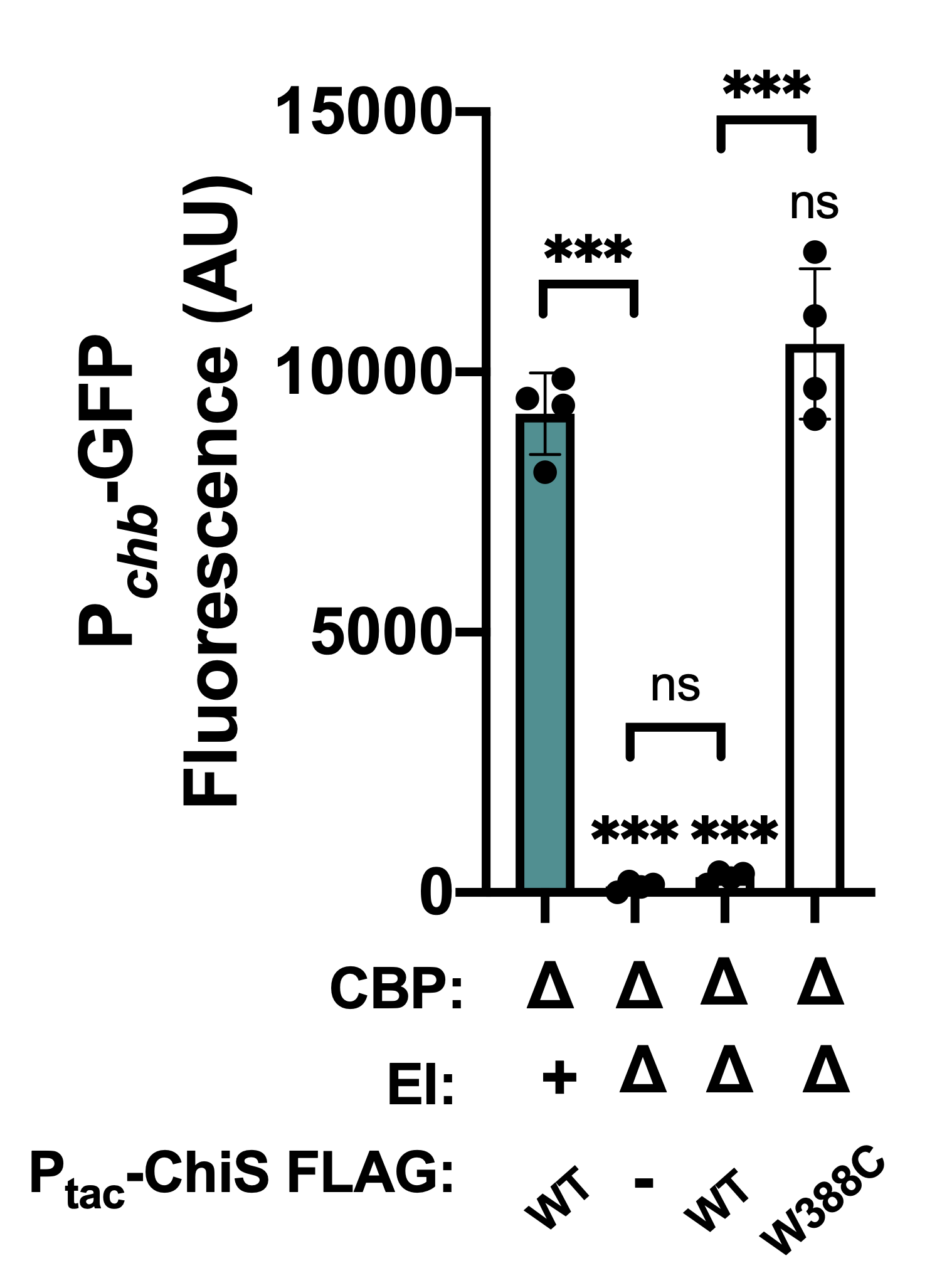

Supplement: S5 Fig — ChiS activation of the chb promoter was assessed in rich medium (in the absence of chitin) using a Pchb-GFP reporter in the indicated strain backgrounds. Strains were grown in the present of 1 μM IPTG to induce ChiS-FLAG constructs. Results are from three independent biological replicates and shown as the mean ± SD. Statistical comparisons were made by one-way ANOVA with Tukey’s multiple comparison test. Statistical identifiers directly above bars represent comparisons to the parent (teal, first bar). NS, not significant. *** = p < 0.001, ** = p < 0.01, * = p < 0.05. (TIFF) [file pgen.1010767.s005.tiff]

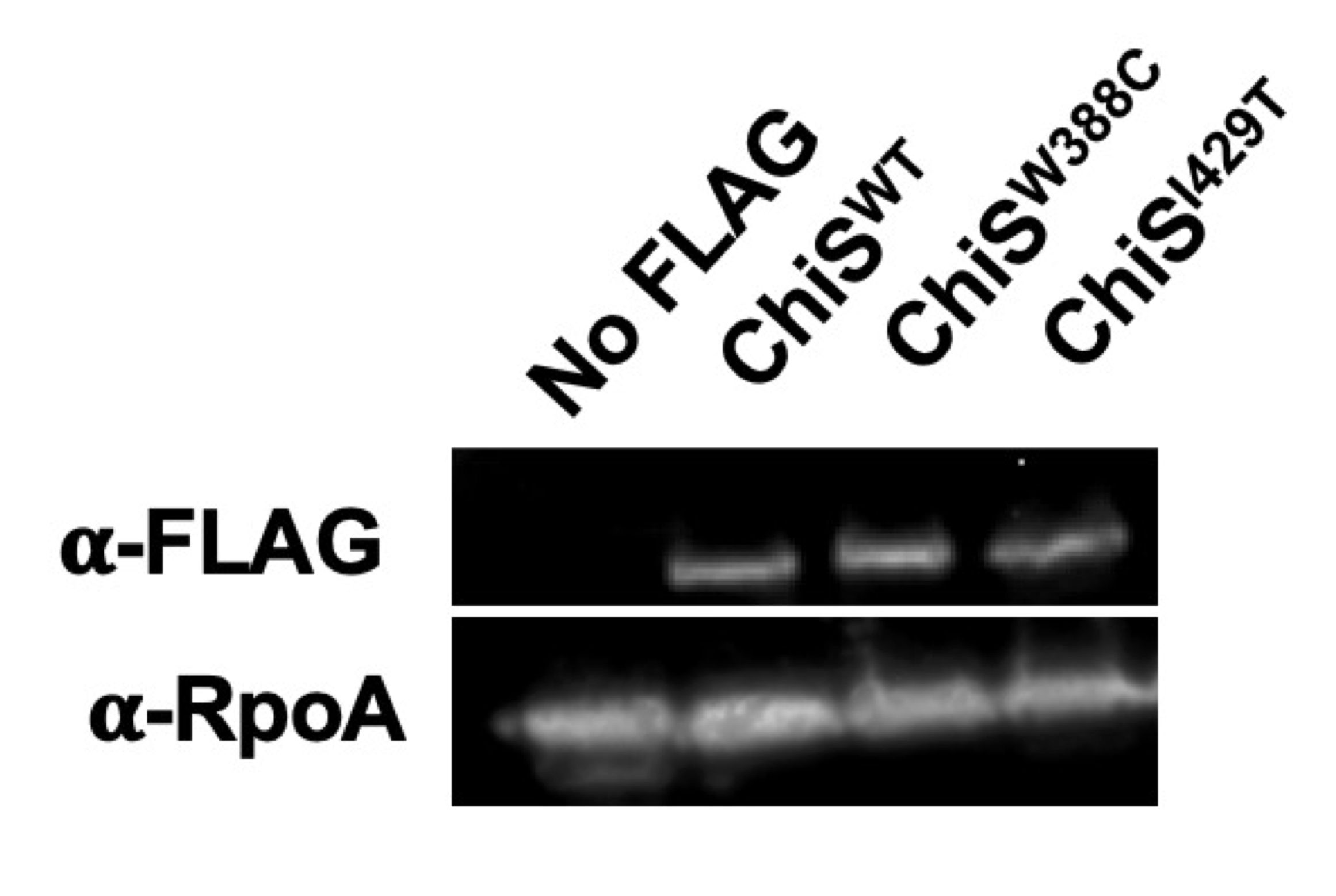

Supplement: S6 Fig — Western blot analysis for ChiS expression. Strains contained a ΔEI mutation and the indicated FLAG-tagged ChiS allele. A strain lacking a FLAG-tagged ChiS allele was also included as a negative control (No FLAG). Blots were developed with primary antibodies against FLAG or RpoA as a loading control as indicated. Data are representative of three independent biological replicates. (TIFF) [file pgen.1010767.s006.tiff]

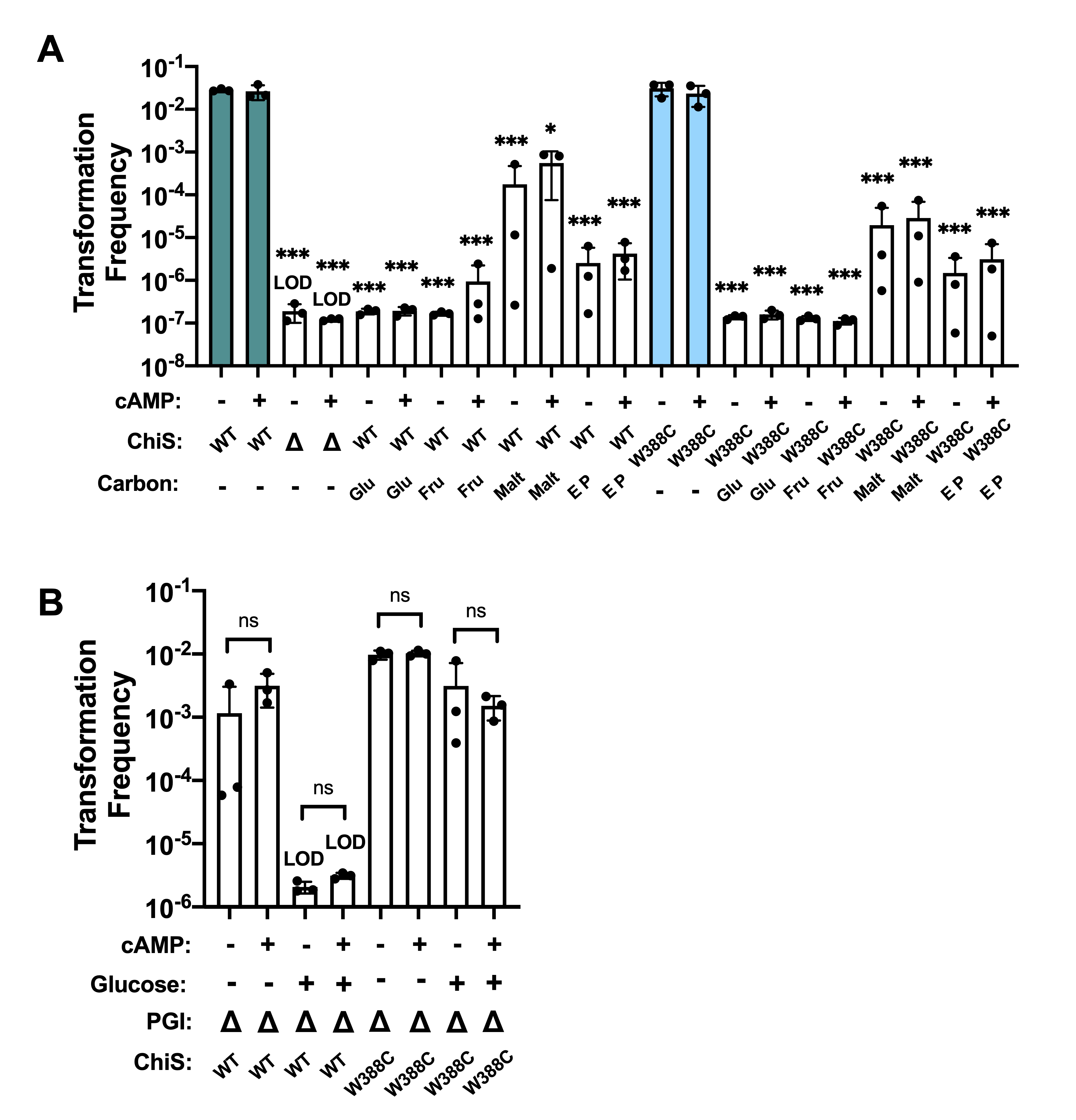

Supplement: S7 Fig — Chitin-induced natural transformation assays of the indicated strains. (A) Transformation reactions were supplemented with 0.5% glucose (Glu), fructose (Fru), maltose (Malt), or a mixture of glutamate and proline (E P) as indicated. (B) Transformation reactions were supplemented with 0.5% glucose as indicated. Transformation reactions were supplemented with 5 mM cAMP as indicated. Results are from three independent biological replicates and shown as the mean ± SD. Statistical comparisons were made by one-way ANOVA with Tukey’s multiple comparison test. NS, not significant. *** = p < 0.001, ** = p < 0.01, * = p < 0.05. LOD, limit of detection. In A, statistics directly above data represent comparison to the parent in equivalent cAMP condition (teal, first two bars and blue, fifth and sixth bars). Data for reactions supplemented with exogenous cAMP in B are identical to that presented in Fig 5 and are included here for ease of comparison. (TIFF) [file pgen.1010767.s007.tiff]

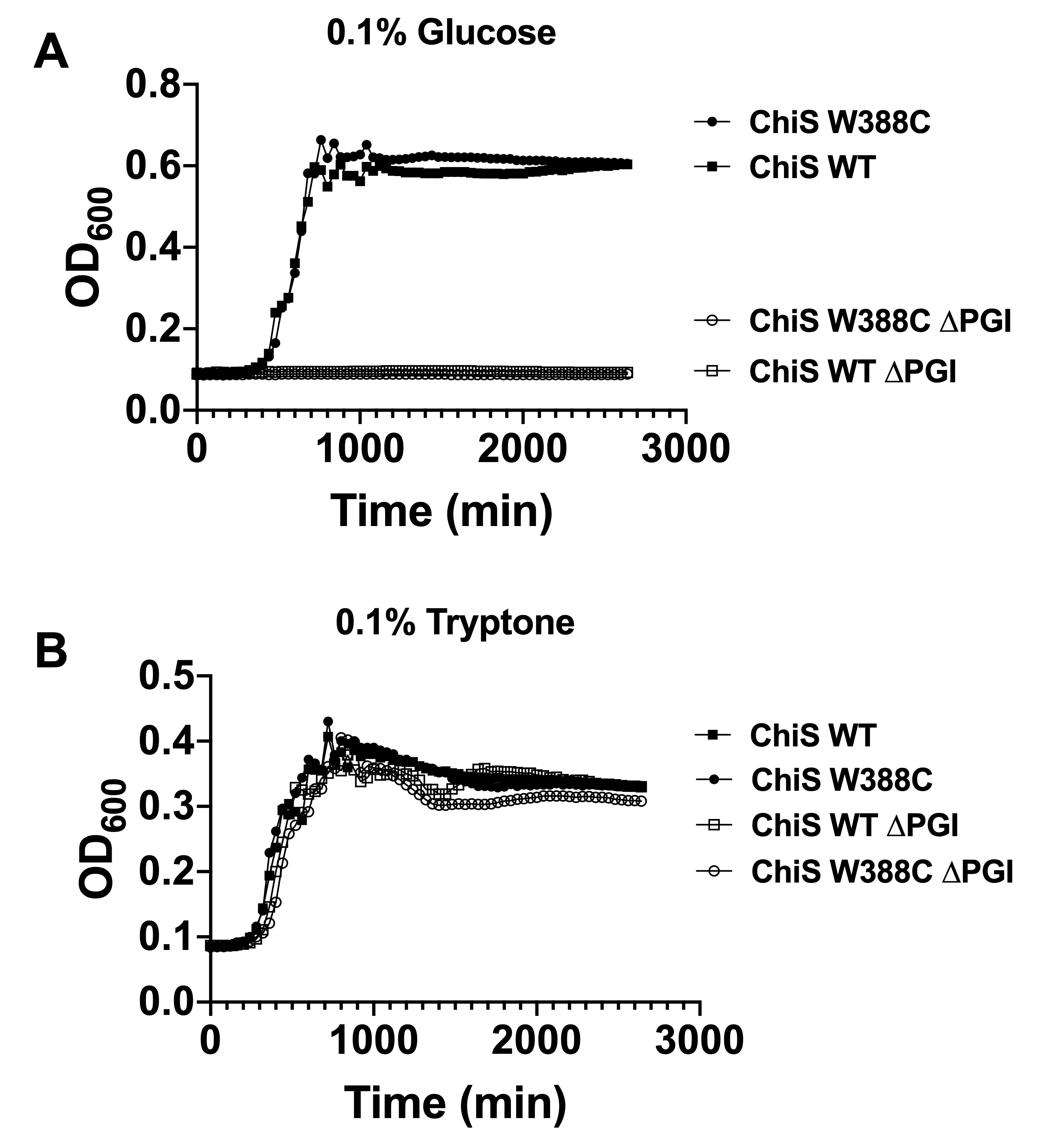

Supplement: S8 Fig — Representative growth curves of the indicated strains in M9 minimal medium containing 0.1% (A) glucose or (B) tryptone as the sole carbon source. Data are representative of three independent biological replicates. (TIFF) [file pgen.1010767.s008.tiff]

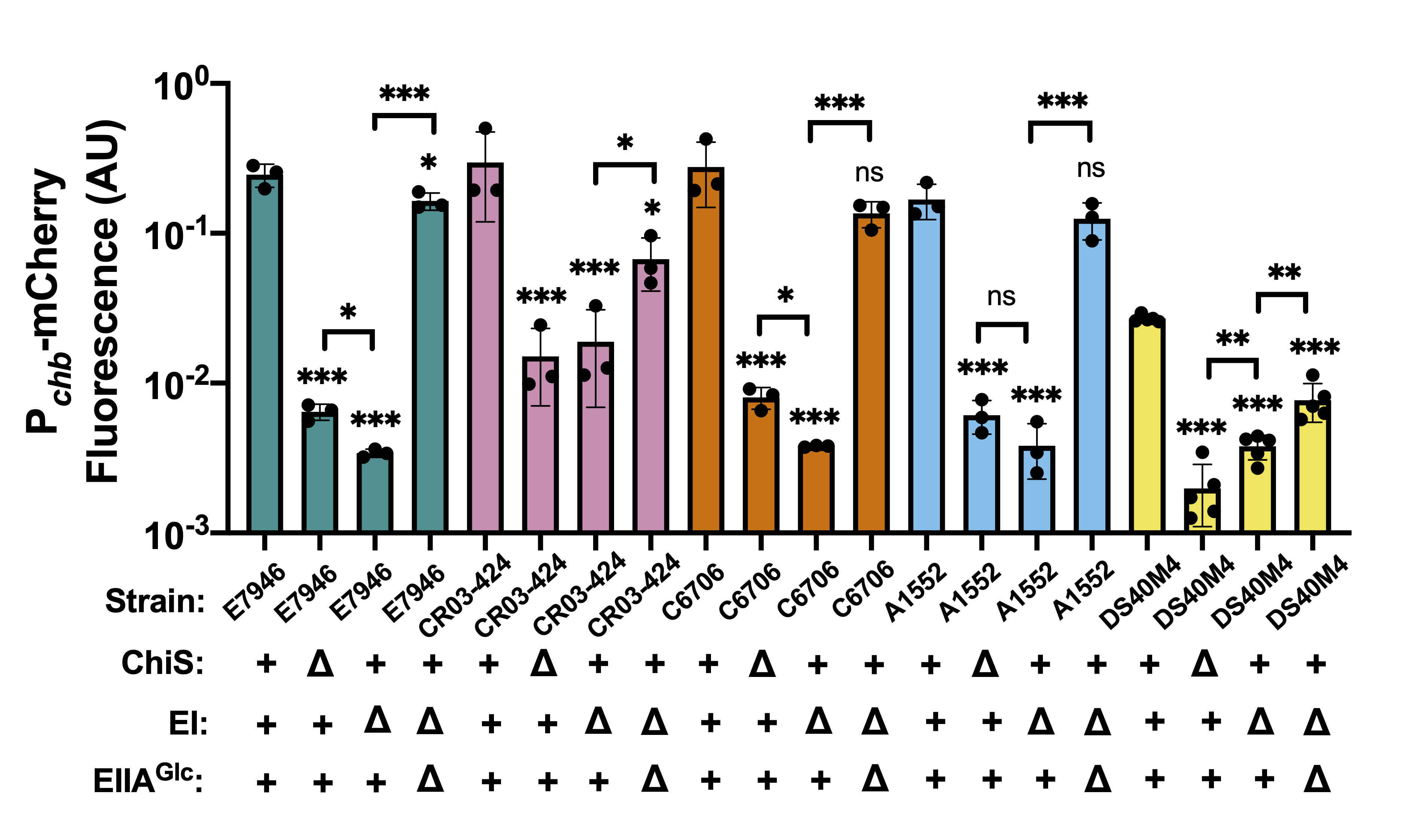

Supplement: S9 Fig — ChiS activation of Pchb-mCherry was assessed in the indicated strains after cells were incubated on chitin for 48 hrs (V. cholerae strains) or 96 hrs (V. campbellii DS40M4) in the presence of 5 mM cAMP. mCherry signal was normalized to a constitutively expressed GFP construct. Each strain is shown in a different color, with V. cholerae E7946 (the parent for all other strains used in this study) shown in teal, the non-toxigenic environmental V. cholerae isolate CR03-424 shown in pink, the toxigenic V. cholerae isolate C6706 shown in orange, the toxigenic V. cholerae isolate A1552 shown in blue, and the V. campbellii DS40M4 strain shown in yellow. Data are from at least three independent biological replicates and are shown as the mean ± SD. Statistical comparisons were made by one-way ANOVA with Tukey’s multiple comparison test. NS, not significant. *** = p < 0.001, ** = p < 0.01, * = p < 0.05. Statistical identifiers directly above bars represent comparisons to the parent of the same strain (first column in each color-coded group). (TIFF) [file pgen.1010767.s009.tiff]
