# Supplementary material for: The molecular mechanism for carbon catabolite repression of the chitin response in Vibrio cholerae
Source: PLoS Genet. 2023 May 12;19(5):e1010767. doi: 10.1371/journal.pgen.1010767 (PMC10208484; doi:10.1371/journal.pgen.1010767)
Supplement: S1 Table — (PDF) [file pgen.1010767.s010.pdf]

**Table S1.** Genes referenced in this study

| Organism | Gene Name / Product | Gene ID |
|---|---|---|
| *V. cholerae* E7946 | *lacZ* / LacZ | VC2338 |
| *V. cholerae* E7946 | *chiS* / ChiS | VC0622 |
| *V. cholerae* E7946 | *cbp* / CBP | VC0620 |
| *V. cholerae* E7946 | *ptsI* / EI | VC0965 |
| *V. cholerae* E7946 | *crr* / $EIIA^{Glc}$ | VC0964 |
| *V. cholerae* E7946 | *cyaA* / CyaA | VC0122 |
| *V. cholerae* E7946 | *pgi* / PGI | VC0374 |
| *V. cholerae* E7946 | *crvA* / CrvA | VCA1075 |
| *V. campbellii* DS40M4 | *chiS* / ChiS | DSB67_12445 |
| *V. campbellii* DS40M4 | *ptsI* / EI | DSB67_03835 |
| *V. campbellii* DS40M4 | *crr* / $EIIA^{Glc}$ | DSB67_03830 |
