# Supplementary material for: The molecular mechanism for carbon catabolite repression of the chitin response in Vibrio cholerae
Source: PLoS Genet. 2023 May 12;19(5):e1010767. doi: 10.1371/journal.pgen.1010767 (PMC10208484; doi:10.1371/journal.pgen.1010767)
Supplement: S2 Table — (PDF) [file pgen.1010767.s011.pdf]

Table S2. Strains used in this study

Strain ID*	Genotype	Reference in Manuscript**
SAD030	*V. cholerae* WT E7946 Sm^{R}	Parent strain for all *V. cholerae* strains in this study (unless otherwise specified)<br><br>Fig. 3D, Fig. 5C, Fig. S6<br><br>No FLAG
SAD3353 / VEG0562	Δ*lacZ*::Kan^{R}-P$_{chb}$-*gfp*; ΔVCA0692::Tm^{R}-P$_{chiS}$-*chiS*; Δ*chiS*::Carb^{R}	Fig. 1A, C-D, Fig. 4A, C-D, Fig. S1A, Fig. S2A-C, Fig. S3, Fig. S7A, Fig. S8A-B<br><br>cbp+ EI+ EIIA^{Glc}+ ChiS WT
SAD3354 / VEG0565	Δ*lacZ*::Kan^{R}-P$_{chb}$-*gfp*; ΔVCA0692::Tm^{R}-P$_{chiS}$-*chiS*; Δ*chiS/cbp*::Carb^{R}	Fig. 1A, Fig. 2, Fig. 3A, Fig. S1A, Fig. S4<br><br>Δcbp EI+ EIIA^{Glc}+ ChiS WT / Untagged ChiS
SAD3355 / VEG0419	Δ*lacZ*::Kan^{R}-P$_{chb}$-*gfp*; ΔVCA0692::Tm^{R}-P$_{chiS}$-*chiS*; Δ*chiS/cbp*::Carb^{R}; Δ*ptsI*	Fig. 1A, Fig. 4A, Fig. S1A, Fig. S4<br><br>Δcbp ΔEI EIIA^{Glc}+ ChiS WT
SAD3356 / VEG0420	Δ*lacZ*::Kan^{R}-P$_{chb}$-*gfp*; ΔVCA0692::Tm^{R}-P$_{chiS}$-*chiS*; Δ*chiS/cbp*::Carb^{R}; Δ*ptsI/crr*::Spec^{R}	Fig. 1A, Fig. 4A, Fig. S1A<br><br>Δcbp ΔEI ΔEIIA^{Glc} ChiS WT
SAD3357 / VEG0816	Δ*lacZ*::Kan^{R}-P$_{chb}$-*gfp*; ΔVCA0692::Tm^{R}-P$_{chiS}$-*chiS*; Δ*chiS/cbp*::Carb^{R}; *crr*^{H91Q}-Spec^{R}	Fig. 1A, Fig. S1A<br><br>Δcbp EI+ EIIA^{Glc} H91Q
SAD3358 / VEG1116	Δ*lacZ*::Kan^{R}-P$_{chb}$-*gfp*; ΔVCA0692::Tm^{R}-P$_{chiS}$-*chiS*; Δ*chiS/cbp*::Carb^{R}; Δ*ptsI*; *crr*^{H91D}-Spec^{R}	Fig. 1A, Fig. S1A<br><br>Δcbp ΔEI EIIA^{Glc} H91D
SAD3359 / VEG0757	Δ*lacZ*::Kan^{R}-P$_{chb}$-*gfp*; ΔVCA0692::Tm^{R}-P$_{chiS}$-*chiS*^{W388C}; Δ*chiS*::Carb^{R}	Fig. 4A, C-D Fig. S1A, Fig. S2A-C, Fig. S3, Fig. S7A, Fig. S8A-B<br><br>cbp+ EI+ EIIA^{Glc}+ ChiS W388C
SAD3360 / VEG0800	Δ*lacZ*::Kan^{R}-P$_{chb}$-*gfp*; ΔVCA0692::Tm^{R}-P$_{chiS}$-*chiS*^{W388C}; Δ*chiS/cbp*::Carb^{R}	Fig. 4A, Fig. S1A<br><br>Δcbp EI+ EIIA^{Glc}+ ChiS W388C
SAD3361 / VEG0741	Δ*lacZ*::Kan^{R}-P$_{chb}$-*gfp*; ΔVCA0692::Tm^{R}-P$_{chiS}$-*chiS*^{W388C}; Δ*chiS/cbp*::Carb^{R}; Δ*ptsI*	Fig. 4A, Fig. S1A<br><br>Δcbp ΔEI EIIA^{Glc}+ ChiS W388C
SAD3362 / VEG1121	Δ*lacZ*::Kan^{R}-P$_{chb}$-*gfp*; ΔVCA0692::Tm^{R}-P$_{chiS}$-*chiS*^{W388C}; Δ*chiS/cbp*::Carb^{R}; Δ*ptsI/crr*::Spec^{R}	Fig. 4A, Fig. S1A<br><br>Δcbp ΔEI ΔEIIA^{Glc} ChiS W388C
SAD3363 / VEG0765	Δ*lacZ*::Kan^{R}-P$_{chb}$-*gfp*; ΔVCA0692::Tm^{R}-P$_{chiS}$-*chiS*^{I429T}; Δ*chiS*::Carb^{R}	Fig. 4A, C-D, Fig. S1A, Fig. S2A-C, Fig. S3<br><br>cbp+ EI+ EIIA^{Glc}+ ChiS I429T
SAD3364 / VEG1052	Δ*lacZ*::Kan^{R}-P$_{chb}$-*gfp*; ΔVCA0692::Tm^{R}-P$_{chiS}$-*chiS*^{I429T}; Δ*chiS/cbp*::Carb^{R}	Fig. 4A, Fig. S1A<br><br>Δcbp EI+ EIIA^{Glc}+ ChiS I429T
SAD3365 / VEG1004	Δ*lacZ*::Kan^{R}-P$_{chb}$-*gfp*; ΔVCA0692::Tm^{R}-P$_{chiS}$-*chiS*^{I429T}; Δ*chiS/cbp*::Carb^{R}; Δ*ptsI*	Fig. 4A, Fig. S1A<br><br>Δcbp ΔEI EIIA^{Glc}+ ChiS I429T
SAD3366 / VEG1122	Δ*lacZ*::Kan^{R}-P$_{chb}$-*gfp*; ΔVCA0692::Tm^{R}-P$_{chiS}$-*chiS*^{I429T}; Δ*chiS/cbp*::Carb^{R}; Δ*ptsI/crr*::Spec^{R}	Fig. 4A, Fig. S1A<br><br>Δcbp ΔEI ΔEIIA^{Glc} ChiS I429T
SAD3367 / VEG1055	Δ*lacZ*::Kan^{R}-P$_{chb}$-*gfp*; ΔVCA0692::Tm^{R}-P$_{chiS}$-*chiS*; Δ*chiS/cbp*::Carb^{R}; Δ*cyaA*	Fig. S1A

		Δcbp EI+ EIIA^{Glc}+ ChiS WT ΔcyaA
SAD3368 / VEG0728	Δ*lacZ*::Kan^R-P$_{chb}$-*gfp*; ΔVCA0692::Tm^R-P$_{chiS}$-*chiS*^{W388C}; Δ*chiS*::Carb^R; Δ*ptsI*	Fig. 4C-D, Fig. S2A-C, Fig. S3<br><br>ΔEI EIIA^{Glc}+ ChiS W388C
SAD3369 / VEG1125	Δ*lacZ*::Kan^R-P$_{chb}$-*gfp*; ΔVCA0692::Tm^R-P$_{chiS}$-*chiS*^{W388C}; Δ*chiS*::Carb^R; Δ*ptsI/crr*::Spec^R	Fig. 4C-D, Fig. S2A-C, Fig. S3<br><br>ΔEI ΔEIIA^{Glc} ChiS W388C
SAD3370 / VEG0736	Δ*lacZ*::Kan^R-P$_{chb}$-*gfp*; ΔVCA0692::Tm^R-P$_{chiS}$-*chiS*^{I429T}; Δ*chiS*::Carb^R; Δ*ptsI*	Fig. 4C-D, Fig. S2A-C, Fig. S3<br><br>ΔEI EIIA^{Glc}+ ChiS I429T
SAD3371 / VEG1126	Δ*lacZ*::Kan^R-P$_{chb}$-*gfp*; ΔVCA0692::Tm^R-P$_{chiS}$-*chiS*^{I429T}; Δ*chiS*::Carb^R; Δ*ptsI/crr*::Spec^R	Fig. 4C-D, Fig. S2A-C, Fig. S3<br><br>ΔEI ΔEIIA^{Glc} ChiS I429T
SAD3372 / VEG1053	Δ*lacZ*::Kan^R-P$_{chb}$-*gfp*; ΔVCA0692::Tm^R-P$_{chiS}$-*chiS*; Δ*chiS*::Carb^R; Δ*cyaA*	Fig. S2A-C, Fig. S3<br><br>EI+ EIIA^{Glc}+ ChiS WT ΔcyaA
SAD3373 / VEG1000	Δ*lacZ*::Kan^R-P$_{chb}$-*mCherry*; igVCA0265-VCA0266::Spec^R-P$_{const2}$-*gfp*; Δ*chiS*::Carb^R	Fig. 1B, Fig. 4B, Fig. S1B, Fig. S9<br><br>EI+ EIIA^{Glc}+ ΔchiS E7946
SAD3374 / VEG0997	Δ*lacZ*::Kan^R-P$_{chb}$-*mCherry*; igVCA0265-VCA0266::Spec^R-P$_{const2}$-*gfp*; ΔVCA0692::Tm^R-P$_{chiS}$-*chiS*; Δ*chiS*::Carb^R	Fig. 1B, Fig. 4B, Fig. S1B<br><br>EI+ EIIA^{Glc}+ ChiS WT
SAD3375 / VEG0974	Δ*lacZ*::Kan^R-P$_{chb}$-*mCherry*; igVCA0265-VCA0266::Spec^R-P$_{const2}$-*gfp*; ΔVCA0692::Tm^R-P$_{chiS}$-*chiS*; Δ*chiS*::Carb^R; Δ*ptsI*	Fig. 1B, Fig. 4B, Fig. S1B<br><br>ΔEI EIIA^{Glc}+ ChiS WT
SAD3376 / VEG0976	Δ*lacZ*::Kan^R-P$_{chb}$-*mCherry*; igVCA0265-VCA0266::Spec^R-P$_{const2}$-*gfp*; ΔVCA0692::Tm^R-P$_{chiS}$-*chiS*; Δ*chiS*::Carb^R; Δ*ptsI/crr*::Erm^R	Fig. 1B, Fig. 4B, Fig. S1B<br><br>ΔEI ΔEIIA^{Glc} ChiS WT
SAD3377 / VEG1124	Δ*lacZ*::Kan^R-P$_{chb}$-*mCherry*; igVCA0265-VCA0266::Spec^R-P$_{const2}$-*gfp*; ΔVCA0692::Tm^R-P$_{chiS}$-*chiS*; Δ*chiS*::Carb^R; *crr*^{H91Q}-Erm^R	Fig. 1B, Fig. S1B<br><br>EI+ EIIA^{Glc} H91Q chiS+
SAD3378 / VEG1118	Δ*lacZ*::Kan^R-P$_{chb}$-*mCherry*; igVCA0265-VCA0266::Spec^R-P$_{const2}$-*gfp*; ΔVCA0692::Tm^R-P$_{chiS}$-*chiS*; Δ*chiS*::Carb^R; Δ*ptsI*; *crr*^{H91D}-Erm^R	Fig. 1B, Fig. S1B<br><br>ΔEI EIIA^{Glc} H91D chiS+
SAD3379 / VEG1058	Δ*lacZ*::Kan^R-P$_{chb}$-*mCherry*; igVCA0265-VCA0266::Spec^R-P$_{const2}$-*gfp*; ΔVCA0692::Tm^R-P$_{chiS}$-*chiS*^{W388C}; Δ*chiS*::Carb^R	Fig. 4B, Fig. S1B<br><br>EI+ EIIA^{Glc}+ ChiS W388C
SAD3380 / VEG0975	Δ*lacZ*::Kan^R-P$_{chb}$-*mCherry*; igVCA0265-VCA0266::Spec^R-P$_{const2}$-*gfp*; ΔVCA0692::Tm^R-P$_{chiS}$-*chiS*^{W388C}; Δ*chiS*::Carb^R; Δ*ptsI*	Fig. 4B, Fig. S1B<br><br>ΔEI EIIA^{Glc}+ ChiS W388C
SAD3381 / VEG0977	Δ*lacZ*::Kan^R-P$_{chb}$-*mCherry*; igVCA0265-VCA0266::Spec^R-P$_{const2}$-*gfp*; ΔVCA0692::Tm^R-P$_{chiS}$-*chiS*^{W388C}; Δ*chiS*::Carb^R; Δ*ptsI/crr*::Erm^R	Fig. 4B, Fig. S1B<br><br>ΔEI ΔEIIA^{Glc} ChiS W388C
SAD3382 / VEG1091	Δ*lacZ*::Kan^R-P$_{chb}$-*mCherry*; igVCA0265-VCA0266::Spec^R-P$_{const2}$-*gfp*; ΔVCA0692::Tm^R-P$_{chiS}$-*chiS*^{I429T}; Δ*chiS*::Carb^R	Fig. 4B, Fig. S1B<br><br>EI+ EIIA^{Glc}+ ChiS I429T
SAD3383 / VEG1090	Δ*lacZ*::Kan^R-P$_{chb}$-*mCherry*; igVCA0265-VCA0266::Spec^R-P$_{const2}$-*gfp*; ΔVCA0692::Tm^R-P$_{chiS}$-*chiS*^{I429T}; Δ*chiS*::Carb^R; Δ*ptsI*	Fig. 4B, Fig. S1B<br><br>ΔEI EIIA^{Glc}+ ChiS I429T
SAD3384 / VEG1146	Δ*lacZ*::Kan^R-P$_{chb}$-*mCherry*; igVCA0265-VCA0266::Spec^R-P$_{const2}$-*gfp*; ΔVCA0692::Tm^R-P$_{chiS}$-*chiS*^{I429T}; Δ*chiS*::Carb^R; Δ*ptsI/crr*::Erm^R	Fig. 4B, Fig. S1B<br><br>ΔEI ΔEIIA^{Glc} ChiS I429T
SAD3385 / VEG1127	Δ*lacZ*::Kan^R-P$_{chb}$-*mCherry*; igVCA0265-VCA0266::Spec^R-P$_{const2}$-*gfp*; ΔVCA0692::Tm^R-P$_{chiS}$-*chiS*; Δ*chiS*::Carb^R; Δ*cyaA*	Fig. S1B<br><br>EI+ EIIA^{Glc}+ ChiS WT ΔcyaA

SAD3386 / VEG0755	Δ*lacZ*::Kan^R-P$_{chb}$-*gfp*; Δ*chiS*::Carb^R	Fig. 1C-D, Fig. 4C-D, Fig. S2A-C, Fig. S3, Fig. S7A<br><br>EI+ EIIA^{Glc}+ ΔchiS
SAD3387 / VEG0753	Δ*lacZ*::Kan^R-P$_{chb}$-*gfp*; ΔVCA0692::Tm^R-P$_{chiS}$-*chiS*; Δ*chiS*::Carb^R; Δ*ptsI*	Fig. 1C-D, Fig. 4C-D, Fig. S2A-C, Fig. S3<br><br>ΔEI EIIA^{Glc}+ ChiS WT
SAD3388 / VEG0754	Δ*lacZ*::Kan^R-P$_{chb}$-*gfp*; ΔVCA0692::Tm^R-P$_{chiS}$-*chiS*; Δ*chiS*::Carb^R; Δ*ptsI/crr*::Spec^R	Fig. 1C-D, Fig. 4C-D, Fig. S2A-C, Fig. S3<br><br>ΔEI ΔEIIA^{Glc} ChiS WT
SAD3389 / VEG0815	Δ*lacZ*::Kan^R-P$_{chb}$-*gfp*; ΔVCA0692::Tm^R-P$_{chiS}$-*chiS*; Δ*chiS*::Carb^R; *crr*^{H91Q}-Spec^R	Fig. 1C-D, Fig. S2A-C, Fig. S3<br><br>EI+ EIIA^{Glc} H91Q chiS+
SAD3390 / VEG1114	Δ*lacZ*::Kan^R-P$_{chb}$-*gfp*; ΔVCA0692::Tm^R-P$_{chiS}$-*chiS*; Δ*chiS*::Carb^R; Δ*ptsI*; *crr*^{H91D}-Spec^R	Fig. 1C-D, Fig. S2A-C, Fig. S3<br><br>EI+ EIIA^{Glc} H91D chiS+
SAD3391 / VEG1059	igVCA0265-VCA0266::Carb^R-P$_{BAD}$-*popZ*; Δ*cbp*::Kan^R; *crr-mCherry*-Spec^R	Fig. 3A-B<br><br>EI+ EIIAGlc+ ChiS-
SAD3392 / VEG1057	igVCA0265-VCA0266::Carb^R-P$_{BAD}$-*popZ*; Δ*cbp*::Kan^R; Δ*ptsI*; *crr-mCherry*-Spec^R	Fig. 3A-B<br><br>ΔEI EIIAGlc+ ChiS-
SAD3393 / VEG0798	igVCA0265-VCA0266::Carb^R-P$_{BAD}$-*popZ*; ΔVCA0692::Tm^R-P$_{tac}$-*chiS-msfGFP-H3H4*; Δ*cbp*::Kan^R; *crr-mCherry*-Spec^R	Fig. 3A-B, Fig. 5A-B<br><br>EI+ EIIA^{Glc}+ ChiS WT
SAD3394 / VEG0799	igVCA0265-VCA0266::Carb^R-P$_{BAD}$-*popZ*; ΔVCA0692::Tm^R-P$_{tac}$-*chiS-msfGFP-H3H4*; Δ*cbp*::Kan^R; Δ*ptsI*; *crr-mCherry*-Spec^R	Fig. 3A-B, Fig. 5A-B<br><br>ΔEI EIIA^{Glc}+ ChiS WT
SAD3395 / VEG1148	igVCA0265-VCA0266::Carb^R-P$_{BAD}$-*popZ*; ΔVCA0692::Tm^R-P$_{tac}$-*chiS*^{W388C}-*msfGFP-H3H4*; Δ*cbp*::Kan^R; *crr-mCherry*-Spec^R	Fig. 5A-B<br><br>EI+ ChiS W388C
SAD3396 / VEG1100	igVCA0265-VCA0266::Carb^R-P$_{BAD}$-*popZ*; ΔVCA0692::Tm^R-P$_{tac}$-*chiS*^{W388C}-*msfGFP-H3H4*; Δ*cbp*::Kan^R; Δ*ptsI*; *crr-mCherry*-Spec^R	Fig. 5A-B<br><br>ΔEI ChiS W388C
SAD3397 / VEG1150	igVCA0265-VCA0266::Carb^R-P$_{BAD}$-*popZ*; ΔVCA0692::Tm^R-P$_{tac}$-*chiS*^{I429T}-*msfGFP-H3H4*; Δ*cbp*::Kan^R; *crr-mCherry*-Spec^R	Fig. 5A-B<br><br>EI+ ChiS I429T
SAD3398 / VEG1136	igVCA0265-VCA0266::Carb^R-P$_{BAD}$-*popZ*; ΔVCA0692::Tm^R-P$_{tac}$-*chiS*^{I429T}-*msfGFP-H3H4*; Δ*cbp*::Kan^R; Δ*ptsI*; *crr-mCherry*-Spec^R	Fig. 5A-B<br><br>ΔEI ChiS I429T
SAD3399 / VEG1026	Δ*lacZ*::Kan^R-P$_{chb}$-*gfp*; ΔVCA0692::Tm^R-P$_{chiS}$-*chiS*; Δ*chiS*::Carb^R; Δ*pgi*::Erm^R	Fig. 6, Fig. S7B, Fig. S8A-B<br><br>ΔPGI ChiS WT
SAD3400 / VEG1031	Δ*lacZ*::Kan^R-P$_{chb}$-*gfp*; ΔVCA0692::Tm^R-P$_{chiS}$-*chiS*^{W388C}; Δ*chiS*::Carb^R; Δ*pgi*::Erm^R	Fig. 5, Fig. S7B, Fig. S8A-B<br><br>ΔPGI ChiS W388C
SAD3401 / VEG1101	Δ*lacZ*::Kan^R-P$_{chb}$-*gfp*; ΔVCA0692::Tm^R-P$_{chiS}$-*chiS* internal 1X FLAG after E566; Δ*chiS/cbp*::Carb^R	Fig. 2<br><br>Δcbp EI+ EIIA^{Glc}+ ChiS WT
SAD3402 / VEG1102	Δ*lacZ*::Kan^R-P$_{chb}$-*gfp*; ΔVCA0692::Tm^R-P$_{chiS}$-*chiS* internal 1X FLAG after E566; Δ*chiS/cbp*::Carb^R; Δ*ptsI*	Fig. 2, Fig. S6<br><br>Δcbp ΔEI EIIA^{Glc}+ ChiS WT
SAD3403 / VEG1123	Δ*lacZ*::Kan^R-P$_{chb}$-*gfp*; ΔVCA0692::Tm^R-P$_{chiS}$-*chiS* internal 1X FLAG after E566; Δ*chiS/cbp*::Carb^R; Δ*ptsI/crr*::Spec^R	Fig. 2<br><br>Δcbp ΔEI ΔEIIA^{Glc} ChiS WT

SAD3404 / VEG1131	Δ*lacZ*::Kan^R-P$_{chb}$-*gfp*; ΔVCA0692::Tm^R-P$_{chiS}$-*chiS*^{W388C} internal 1X FLAG after E566; Δ*chiS/cbp*::Carb^R	Fig. 2<br><br>Δcbp EI+ EIIA^{Glc}+ ChiS W388C
SAD3405 / VEG1132	Δ*lacZ*::Kan^R-P$_{chb}$-*gfp*; ΔVCA0692::Tm^R-P$_{chiS}$-*chiS*^{W388C} internal 1X FLAG after E566; Δ*chiS/cbp*::Carb^R; Δ*ptsI*	Fig. 2, Fig. S6<br><br>Δcbp ΔEI EIIA^{Glc}+ ChiS W388C
SAD3406 / VEG1099	Δ*lacZ*::Kan^R-P$_{chb}$-*gfp*; ΔVCA0692::Tm^R-P$_{chiS}$-*chiS*^{I429T} internal 1X FLAG after E566; Δ*chiS/cbp*::Carb^R	Fig. 2<br><br>Δcbp EI+ EIIA^{Glc}+ ChiS I429T
SAD3407 / VEG1134	Δ*lacZ*::Kan^R-P$_{chb}$-*gfp*; ΔVCA0692::Tm^R-P$_{chiS}$-*chiS*^{I429T} internal 1X FLAG after E566; Δ*chiS/cbp*::Carb^R; Δ*ptsI*	Fig. 2, Fig. S6<br><br>Δcbp ΔEI EIIA^{Glc}+ ChiS I429T
SAD3408 / VEG1133	Δ*lacZ*::Kan^R-P$_{chb}$-*gfp*; ΔΔ*chiS/cbp*::Carb^R; ΔVCA0692::Tm^R-P$_{chiS}$-*chiS*; *crr-mCherry*-Spec^R	Fig. S4<br><br>Δcbp EI+ EIIA^{Glc}-mCherry
SAD3409 / VEG0797	Δ*lacZ*::Kan^R-P$_{chb}$-*gfp*; Δ*chiS/cbp*::Carb^R; ΔVCA0692::Tm^R-P$_{chiS}$-*chiS*; Δ*ptsI*; *crr-mCherry*-Spec^R	Fig. S4<br><br>Δcbp ΔEI EIIA^{Glc}-mCherry
SAD3428 / VEG1220	igVCA0265-VCA0266::Carb^R-P$_{BAD}$-*popZ*; ΔVCA0692::Tm^R-P$_{tac}$-*chiS-msfGFP-H3H4*; Δ*cbp*::Kan^R; Δ*ptsI*; *crr-mCherry*-Spec^R; Δ*crvA*::Zeo^R	Fig. 3C
SAD3442 / VEG1184	ΔVCA0692::Tm^R-P$_{tac}$-*chiS*^{I429T}*-msfGFP-H3H4*; Δ*ptsI*; *crr-mCherry*-Spec^R; Δ*cbp*::Kan^R; igVCA0265-VCA0266::Carb^R-P$_{BAD}$-*popZ*	Fig. 5A-B<br><br>ΔEI ChiS W388C
SAD3443 / VEG0801	Δ*cbp*::Kan^R; *crr*^{H91Q}*-mCherry*-Spec^R ΔVCA0692::Tm^R-P$_{tac}$-*chiS-msfGFP-H3H4*; igVCA0265-VCA0266::Carb^R-P$_{BAD}$-*popZ*	Fig. 3A-B<br><br>EI+ EIIA^{Glc} H91Q ChiS+
SAD3444 / VEG0804	Δ*cbp*::Kan^R; Δ*ptsI*; *crr*^{H91D}*-mCherry*-Spec^R; ΔVCA0692::Tm^R-P$_{tac}$-*chiS-msfGFP-H3H4*; igVCA0265-VCA0266::Carb^R-P$_{BAD}$-*popZ*	Fig. 3A-B<br><br>ΔEI EIIA^{Glc} H91D ChiS+
SAD3445 / VEG1303	Δ*ptsI*; *crr-mCherry*-Spec^R; Δ*chiS/cbp*::Carb^R; pMMB67EH *tfoX qstR* Cm^R; Δ*lacZ*::Kan^R-P$_{chb}$-*gfp*	Fig. 3D, Fig. 5C, Fig. S5<br><br>ΔCBP ΔEI ChiS -
SAD3446 / VEG1301	*crr-mCherry*-Spec^R; Δ*lacZ*::Kan^R-P$_{chb}$-*gfp*; ΔVCA0692::Tm^R-P$_{tac}$-*chiS* internal 1X FLAG after E566; Δ*chiS/cbp*::Carb^R	Fig. 3D, Fig. 5C, Fig. S5<br><br>ΔCBP EI+ ChiS WT
SAD3447 / VEG1288	Δ*ptsI*; *crr-mCherry*-Spec^R; Δ*lacZ*::Kan^R-P$_{chb}$-*gfp*; ΔVCA0692::Tm^R-P$_{tac}$-*chiS* internal 1X FLAG after E566; Δ*chiS/cbp*::Carb^R	Fig. 3D, Fig. 5C, Fig. S5<br><br>ΔCBP ΔEI ChiS WT
SAD3448 / VEG1321	Δ*ptsI*; *crr-mCherry*-Spec^R; Δ*lacZ*::Kan^R-P$_{chb}$-*gfp*; ΔVCA0692::Tm^R-P$_{tac}$-*chiS*^{W388C} internal 1X FLAG after E566; Δ*chiS/cbp*::Carb^R	Fig. 5C, Fig. S5<br><br>ΔCBP ΔEI ChiS W388C
SAD3449 / VEG1289	igVCA0265-VCA0266::Spec^R-P$_{const2}$-*gfp*; Δ*lacZ*::Kan^R-P$_{chb}$-*mCherry*	Fig. S9<br><br>E7946 ChiS+ EI+ EIIA^{Glc}+
SAD3450 / VEG1290	Δ*ptsI*::Erm^R; igVCA0265-VCA0266::Spec^R-P$_{const2}$-*gfp*; Δ*lacZ*::Kan^R-P$_{chb}$-*mCherry*	Fig. S9<br><br>E7946 ChiS+ ΔEI EIIA^{Glc}+
SAD3451 / VEG1291	Δ*ptsI/crr*::Erm^R; igVCA0265-VCA0266::Spec^R-P$_{const2}$-*gfp*; Δ*lacZ*::Kan^R-P$_{chb}$-*mCherry*	Fig. S9<br><br>E7946 ChiS+ ΔEI ΔEIIA^{Glc}

SAD3452 / VEG1322	*V. cholerae* CR03-424<br><br>Δ*chiS*::Carb^R; ΔVCA0692::Spec^R-P_{const2}-*gfp*; Δ*lacZ*::Kan^R-P_{chb}-*mCherry*	Fig. S9<br><br>CR03-424 ΔchiS EI+ $EIIA^{Glc}$+
SAD3453 / VEG1262	*V. cholerae* CR03-424<br><br>ΔVCA0692::Spec^R-P_{const2}-*gfp*; Δ*lacZ*::Kan^R-P_{chb}-*mCherry*	Fig. S9<br><br>CR03-424 ChiS+ EI+ $EIIA^{Glc}$+
SAD3454 / VEG1269	*V. cholerae* CR03-424<br><br>Δ*ptsI*::Erm^R; ΔVCA0692::Spec^R-P_{const2}-*gfp*; Δ*lacZ*::Kan^R-P_{chb}-*mCherry*	Fig. S9<br><br>CR03-424 ChiS+ ΔEI $EIIA^{Glc}$+
SAD3455 / VEG1271	*V. cholerae* CR03-424<br><br>Δ*ptsI/crr*::Erm^R; ΔVCA0692::Spec^R-P_{const2}-*gfp*; Δ*lacZ*::Kan^R-P_{chb}-*mCherry*	Fig. S9<br><br>CR03-424 ChiS+ ΔEI Δ$EIIA^{Glc}$
SAD3456 / VEG1265	*V. cholerae* C6706<br><br>Δ*chiS*::Carb^R; igVCA0265-VCA0266::Spec^R-P_{const2}-*gfp*; Δ*lacZ*::Kan^R-P_{chb}-*mCherry*	Fig. S9<br><br>C6706 ΔchiS EI+ $EIIA^{Glc}$+
SAD3457 / VEG1250	*V. cholerae* C6706<br><br>igVCA0265-VCA0266::Spec^R-P_{const2}-*gfp*; Δ*lacZ*::Kan^R-P_{chb}-*mCherry*	Fig. S9<br><br>C6706 ChiS+ EI+ $EIIA^{Glc}$+
SAD3458 / VEG1255	*V. cholerae* C6706<br><br>Δ*ptsI*::Erm^R; igVCA0265-VCA0266::Spec^R-P_{const2}-*gfp*; Δ*lacZ*::Kan^R-P_{chb}-*mCherry*	Fig. S9<br><br>C6706 ChiS+ ΔEI $EIIA^{Glc}$+
SAD3459 / VEG1259	*V. cholerae* C6706<br><br>Δ*ptsI/crr*::Erm^R; igVCA0265-VCA0266::Spec^R-P_{const2}-*gfp*; Δ*lacZ*::Kan^R-P_{chb}-*mCherry*	Fig. S9<br><br>C6706 ChiS+ ΔEI Δ$EIIA^{Glc}$
SAD3460 / VEG1266	*V. cholerae* A1552<br><br>Δ*chiS*::Carb^R; igVCA0265-VCA0266::Spec^R-P_{const2}-*gfp*; Δ*lacZ*::Kan^R-P_{chb}-*mCherry*	Fig. S9<br><br>A1552 ΔchiS EI+ $EIIA^{Glc}$+
SAD3461 / VEG1251	*V. cholerae* A1552<br><br>igVCA0265-VCA0266::Spec^R-P_{const2}-*gfp*; Δ*lacZ*::Kan^R-P_{chb}-*mCherry*	Fig. S9<br><br>A1552 ChiS+ EI+ $EIIA^{Glc}$+
SAD3462 / VEG1256	*V. cholerae* A1552<br><br>Δ*ptsI*::Erm^R; igVCA0265-VCA0266::Spec^R-P_{const2}-*gfp*; Δ*lacZ*::Kan^R-P_{chb}-*mCherry*	Fig. S9<br><br>A1552 ChiS+ ΔEI $EIIA^{Glc}$+
SAD3463 / VEG1260	*V. cholerae* A1552<br><br>Δ*ptsI/crr*::Erm^R; igVCA0265-VCA0266::Spec^R-P_{const2}-*gfp*; Δ*lacZ*::Kan^R-P_{chb}-*mCherry*	Fig. S9<br><br>A1552 ChiS+ ΔEI Δ$EIIA^{Glc}$
SAD3464 / VEG1300	*V. campbellii* DS40M4<br><br>pMMB67EH *tfoX* Gent^R	DS40M4<br><br>Parent for all *V. campbellii* strains used in this study
SAD3465 / VEG1315	*V. campbellii* DS40M4	Fig. S9<br><br>DS40M4 ΔchiS EI+ $EIIA^{Glc}$+

	Δ*chiS*::Erm^{R}; igDSB10880_10885::Spec^{R}-P$_{const2}$-*gfp*; igDSB08575_08580::Kan^{R}-P$_{chb}$-*mCherry*; pMMB67EH *tfoX* Gent^{R}	
SAD3466 / VEG1320	*V. campbellii* DS40M4<br><br>igDSB10880_10885::Spec^{R}-P$_{const2}$-*gfp*; igDSB08575_08580::Kan^{R}-P$_{chb}$-*mCherry*; pMMB67EH *tfoX* Gent^{R}	Fig. S9<br><br>DS40M4 ChiS+ EI+ EIIA^{Glc}+
SAD3467 / VEG1316	*V. campbellii* DS40M4<br><br>Δ*ptsI*::Erm^{R}; igDSB10880_10885::Spec^{R}-P$_{const2}$-*gfp*; igDSB08575_08580::Kan^{R}-P$_{chb}$-*mCherry*; pMMB67EH *tfoX* Gent^{R}	Fig. S9<br><br>DS40M4 ChiS+ ΔEI EIIA^{Glc}+
SAD3468 / VEG1317	*V. campbellii* DS40M4<br><br>Δ*ptsI/crr*::Erm^{R}; igDSB10880_10885::Spec^{R}-P$_{const2}$-*gfp*; igDSB08575_08580::Kan^{R}-P$_{chb}$-*mCherry*; pMMB67EH *tfoX* Gent^{R}	Fig. S9<br><br>DS40M4 ChiS+ ΔEI ΔEIIA^{Glc}

**Double identifiers under 'Strain ID' refer to the same strain that has been stocked in two independent strain collections.*

***'Reference in Manuscript' describes the figures in which this strain is found and the relevant genotype used in those figures to refer to the strain.*
