# Supplementary material for: The molecular mechanism for carbon catabolite repression of the chitin response in Vibrio cholerae
Source: PLoS Genet. 2023 May 12;19(5):e1010767. doi: 10.1371/journal.pgen.1010767 (PMC10208484; doi:10.1371/journal.pgen.1010767)
Supplement: S3 Table — (PDF) [file pgen.1010767.s012.pdf]

Table S3. Primers used in this study

Primers for SOE deletions		
**Primer**	**Sequence**	**Description**
ABD123	ATTCCGGGGATCCGTCGAC	Antibiotic resistance cassette F
ABD124	TGTAGGCTGGAGCTGCTTC	Antibiotic resistance cassette R
ABD767	TTAATTTGGATCCCTGCGACACTC	ΔchiS F1 for Up arm
ABD768	gtcgacggatccccggaatCAAAAAACGTGAGGAGA ATGCC	ΔchiS R1 for Up arm
ABD769	gaagcagctccagcctacaTTCTTGAGCATTGCAAAG AAGC	ΔchiS F2 for Down arm
ABD770	CTGGAACGAATGAAGAAGTCCAG	ΔchiS R2 for Down arm
VGP0199	TCAAACAAAGGCTGACTGAATC	ΔchiS detect R
ABD796	TTAGAATCTGCGCCAGAAGCG	Δcbp F1 for Up arm
ABD797	gtcgacggatccccggaatCATAGCTGTTCCTTACTA GTTGC	Δcbp R1 for Up arm
ABD798	gaagcagctccagcctacaGTACTGGATCTGAAACC AGTTAAG	Δcbp F2 for Down arm
ABD799	GTATTGCGGAATGACCAGCATG	Δcbp R2 for Down arm
VGP0200	GAACAGTGACCAACCAAATGC	Δcbp detect R
ABD963	GCTTGGATGCTGGCTATACAG	ΔEI F1 for Up arm
ABD966	gtcgacggatccccggaatCATAGCCTTACCTTAAACTG GTC	ΔEI R1 for Up arm
ABD967	gaagcagctccagcctacaTAATTATCGGTTGATACCA AGGAG	ΔEI F2 for Down arm
ABD968	TTGATTAATGTGCAGACGGAGC	ΔEI R2 for Down arm
VGP0096	GATAGTACCGTTAACAGGAGCTAC	ΔEI detect R
VGP0001	TGATTGATCGTGTTGAGCGTG	ΔEIIAGlc F1 for Up arm
BBC066	gtcgacggatccccggaatCATTGTGTCATGCTCCTAA CG	ΔEIIAGlc R1 for Up arm
BBC067	gaagcagctccagcctacaACCAAGTAATCGCTTGGTT CG	ΔEIIAGlc F2 for Down arm
BBC068	GGTCGCGATACGCAGATTC	ΔEIIAGlc R2 for Down arm
VGP0100	CGTCATGCTCAGCAGTATGTG	ΔEIIAGlc detect R
BBC040	TTGATCGCCGTTTCTAACTTGG	ΔcyaA F1 for Up arm
BBC041	gtcgacggatccccggaatCAAGTTTGCTTCCCTGATAT G	ΔcyaA R1 for Up arm
BBC042	gaagcagctccagcctacaTAACTCGTTGACGTCTCAG G	ΔcyaA F2 for Down arm
BBC043	CTTGCAGCGACGAGCATTTTAG	ΔcyaA R2 for Down arm
BBC044	CTGGCTGGCCTCCAAATCAGG	ΔcyaA detect R
BBC1492	AGTGCTGCCAACATTAATGCC	Δpgi F1 for Up arm
BBC1493	gtcgacggatccccggaatCATGGCATCTATCCCGATG	Δpgi R1 for Up arm
BBC1494	gaagcagctccagcctacaTAATTACGCCGAATAACCA ATTG	Δpgi F2 for Down arm
BBC1495	GTTGTACGCCAGAAAATCGCC	Δpgi R2 for Down arm
BBC1496	TAGCGTTGATCACTTTGCC	Δpgi detect R
BBC1782	CAAGCGATGTACGAACGAAC	ΔcrvA F1 for Up arm
BBC1783	gtcgacggatccccggaatCCACATAAAGTGGGAAAGA CAAAC	ΔcrvA R1 for Up arm
BBC1784	gaagcagctccagcctacaGTCAATGGCAATGACACG G	ΔcrvA F2 for Down arm
BBC1785	GCTTGACGTTGTTGCTTACTGG	ΔcrvA R2 for Down arm

BBC1786	TTTTGTGATGGCTGGATGTCG	ΔcrvA detect R
ABD725	GAAGCAGCTCCAGCCTACA	Detect F for all deletions
VGP0305	ACCAAGAAACCGTAACGTGCTAC	V. campbellii ΔchiS F1 for Up arm
VGP0306	gtcgacggatccccggaatCATTAGCGTGCTTTGCAGTCG	V. campbellii ΔchiS R1 for Up arm
VGP0307	gaagcagctccagcctacaTAACGCAAACCACCAAATATCAG	V. campbellii ΔchiS F2 for Down arm
VGP0308	GATAGTACAACTGCAGTACCTGC	V. campbellii ΔchiS R2 for Down arm
VGP0309	CTAGGTGAAATTTAATCTGCCG	V. campbellii ΔchiS detect R
VGP0295	TTTATCCTCTTGGTCAACGTCC	V. campbellii ΔEI or ΔEI/EIIAGlc F1 for Up arm
VGP0296	gtcgacggatccccggaatCATAGCCTTACCTTAAGTTGGTC	V. campbellii ΔEI or ΔEI/EIIAGlc R1 for Up arm
VGP0297	gaagcagctccagcctacaTAATCAACTAATTCCAATGAGATAGG	V. campbellii ΔEI F2 for Down arm
VGP0298	CTAAACAACGCAAACTCAGCAC	V. campbellii ΔEI R2 for Down arm
VGP0299	GCCTTTCAGTTCAACAGTGTCG	V. campbellii ΔEI detect R
VGP0302	gaagcagctccagcctacaACTCCAGTTCTACGTGTAACTAAG	V. campbellii ΔEI/EIIAGlc F2 for Down arm
VGP0303	ACTATTGCAGTAGTTTACCTAGCC	V. campbellii ΔEI/EIIAGlc R2 for Down arm
VGP0304	TTAGCGGATGAGCTTGACGTTAC	V. campbellii ΔEI/EIIAGlc detect R
**Primers for point mutations**		
**Primer**	**Sequence**	**Description**
VGP0105	CTTTTGAAGCTGACATGtGCAGCCGAACGTGTTTC	ChiS W388C F
VGP0106	GAAACACGTTCGGCTGCaCATGTCAGCTTCAAAAG	ChiS W388C R
VGP0144	CATTCGCGAAAAAAccTCGCTGATCAAATCGC	ChiS I429T F
VGP0145	GCGATTTGATCAGCGAggTTTTTTCGCGAATG	ChiS I429T R
VGP0006	CTGTTTGTTCAaTTCGGTATCG	EIIAGlc H91Q F
VGP0012	CGATACCGAAtTGAACAAACAG	EIIAGlc H91Q R
VGP0002	CTGTTTGTTgAtTTCGGTATCGAC	EIIAGlc H91D F
VGP0003	GTCGATACCGAAaTcAACAAACAG	EIIAGlc H91D R
**Primers for ChIP-qPCR**		
**Primer**	**Sequence**	**Description**
BBC989	GCATCTAGGTTTTGACGTTTTTAACG	Pchb F
BBC990	AACACTCTCCAAGACCTACCTC	Pchb R
ABD132	CTGTCTCAAGCCGGTTACAA	rpoB F
ABD133	TTTCTACCAGTGCAGAGATGC	rpoB R
**Primers for reporter constructs**		
**Primer**	**Sequence**	**Description**
VGP0093	tccaccacttccacctgcCTTGGTCACGCGTAGGATCGG	EIIAGlc-C terminal tag R1
VGP0094	gcaggtggagcaggtggaTAATCGCTTGGTTCGATTTAAAAC	EIIAGlc-C terminal tag F2
BBC3222	GCaGGTGGAAGTGGTGGAgtgagcaagggcgaggaggataacatgg	mCherry (middle arm) F
BBC3223	tccacctgctccacctgccttgtacagctcgtccatgccgccggtggag	mCherry (middle arm) R

BBC3230	GCAGTAAATCCGACTTTGGAG	Insert construct at igVCA0265-VCA0266 F1
CKP802	caccatacccacgccgaaacaaAGCATTCTGTTGTTATGCTG	Insert construct at igVCA0265-VCA0266 R1
CKP803	gaagcagctccagcctacaTCATAATTTAAGGCGTTAGCAG	Insert construct at igVCA0265-VCA0266 F2
BBC3231	TCTCATAAACCTCATGGTAGACG	Insert construct at igVCA0265-VCA0266 R2
BBC252	caatttcacacaggatcccgggaggaggtaacgtaATGCGTAAAGGAGAAGAAC	Amplify GFP for inserting after Pconst2 F
BBC254	tgtaggctggagctgcttcTTAGTTGTATAGTTCATCCATGCC	Amplify GFP for inserting after Pconst2 at igVCA0265-VCA0266 or VCA0602 R
BBC3082	ttatacgagccttatgcatgcccgtaaagttatccagcaaccactcatagacctagggcagcagatagggacgacgtggtgttagctgtgCTCATTAGGCACCCCAGGC	Insert Pconst2 promoter upstream of GFP R1
BBC3083	tggataactttacgggcatgcataaggctcgtataatatattcagggagaccacaacggtttccctctacaaataattttgtttaactttCAATTTCACACAGGATCCCGGG	Insert Pconst2 promoter upstream of GFP F2
VGP0281	GGAATGACTATCTCGTAATGGAGAC	Insert construct at *V. campbellii* igDSB67_10880-10885 F1
VGP0282	caccatacccacgccgaaacaaACTTGTTGTGCATTGGAATAACTTG	Insert construct at *V. campbellii* igDSB67_10880-10885 R1
VGP0283	gaagcagctccagcctacaAGACCCATAGCCCAGCAACAAG	Insert construct at *V. campbellii* igDSB67_10880-10885 F2
VGP0284	GAAGTTATCAGTTAGAGCGATACC	Insert construct at *V. campbellii* igDSB67_10880-10885 R2
VGP0287	AAGAAGGTACATTATGGACGCAG	Detect construct at *V. campbellii* igDSB67_10880-10885 R
VGP0288	CAACCTAGTTCAGAGCAAACTTG	Insert construct at *V. campbellii* igDSB67_08575-08580 F1
VGP0289	caccatacccacgccgaaacaaTGATCATGATGTCTTAGAGCGC	Insert construct at *V. campbellii* igDSB67_08575-08580 R1
VGP0290	gaagcagctccagcctacaGCCTCTTAGTTAATCAGCTGAG	Insert construct at *V. campbellii* igDSB67_08575-08580 F2
VGP0291	TATGGTAAGACGCTTGCTCCAC	Insert construct at *V. campbellii* igDSB67_08575-08580 R2
VGP0292	TAGTTAACCCTGCGTTTGGTTG	Detect construct at *V. campbellii* igDSB67_08575-08580 R
