# Supplementary material for: The molecular mechanism for carbon catabolite repression of the chitin response in Vibrio cholerae
Source: PLoS Genet. 2023 May 12;19(5):e1010767. doi: 10.1371/journal.pgen.1010767 (PMC10208484; doi:10.1371/journal.pgen.1010767)
Supplement: Attachment — Submitted filename: Response to Reviewers PLoS Genetics final.docx [file pgen.1010767.s014.docx]

**We would like to thank the reviewers for their constructive suggestions on our initial submission. We have addressed all of the comments and concerns that were raised by performing extensive additional experiments and by modifying the text as suggested. Two of the major experimental additions to the manuscript include (1) biochemical work to support an interaction between ChiS and EIIA^Glc^, and (2) assays that demonstrate that the mechanism of CCR described here is conserved in diverse strains of *V. cholerae* as well as a more distantly related *Vibrio* species (*V. campbellii* DS40M4). We believe that these changes have greatly strengthened our manuscript’s claims and broader significance. See below for our responses to individual comments, shown in bold. We have also provided a marked-up version of the manuscript where all changes to the main text are shown in red.**

Reviewer #1: This is nicely detailed genetics work from the Dalia group that demonstrates the molecular details behind carbon catabolite repression on the utilization of chitin polymer and competence for DNA uptake in Vibrio cholerae. Initiated by previous studies, they here dissect the proteins (EIIA-Glc and ChiS) involved in connecting CCR and chitin-induced regulation. The molecular details are appreciated and the numerous alternative approaches demonstrate the role of specific protein-protein interaction. The experiments seem well-controlled and properly explained. Only minor comments.

The term evolution (line 18, 28, 44, 310) is too strong here, as the exchange of genetic material does not equal evolution (=mutations/genetic variance + selection/adaptation). The authors should rather refer to genetic exchange or HGT, but not evolution directly.

**We have amended the text.**

Line 46: Another disadvantage of excreted enzymes, like chitinase of Vibrio is the exploitability, which could be mentioned in this sentence (see <https://doi.org/10.1016/j.cub.2013.10.030>).

**We have included the potential for losing secreted enzymes in our discussion of the energetic cost of chitin metabolism.**

Line 78: introduce what “dephospho-” means. This becomes clear at the results section, but should be clear also here for the readers.

**We have explained this abbreviation.**

Line 387: include respective filter excitation and emission wavelength ranges

**We have added this information.**

Statistics are lacking in the figures, which should be added for all figures, but becomes especially relevant for Fig 1C, where some of the differences are not as huge as in other panels. Nevertheless, even if the differences are high, significance should be added for all. Also, the data points would be preferable to include for each bar graph, which is becoming a standard for the field (see example: <https://www.nature.com/articles/s41551-017-0079>).

**We have added the individual data points to all bar graphs and performed statistical analyses as suggested. Relevant statistical comparisons (made by one-way ANOVAs with Tukey’s multiple comparisons test) are denoted directly in the figures. A spreadsheet of summary statistics for all data are now included in S1 Dataset.**

Reviewer #2: In this manuscript, the authors delineate a novel mechanisms by which carbon catabolite repression of chitin-inducible gene expression might occur in Vibrio. The work is interesting in that it points to a causal involvement of EIIA-Glc. At the same time, the work is firmly rooted in previously published data (ref 22 and others), and lacks some of the data that are needed to support the claim of a direct interaction (as posed in Figure 7). Overall, I feel that the manuscript would benefit from further experimentation as well as a critical description of the results.

Major points:
1. The authors do not address whether this is a conserved response. With large (serogroup) variety, it would be important to know whether the observed effects are broadly conserved or limited to a specific strain of V. cholera. This type of broad impact is what I would expect for a PGEN manuscript.

**As the reviewer suggests, these results would be more compelling if the response were assessed among diverse strains of *V. cholerae*. We therefore tested the ability of dephosphorylated EIIA^Glc^ to repress ChiS transcriptional activity in our chitin-induced reporter assay in two other toxigenic *V. cholerae* strains (C6706, A1552), a nontoxigenic, non-O1/O139 environmental isolate of *V, cholerae* (CR03-424), and a more distantly related species - *Vibrio campbellii* DS40M4. In all strains tested, we observed a ChiS-dependent activation of the P*_chb_*-*mCherry* reporter in response to chitin which could be repressed by the deletion of EI and rescued by the tandem deletion of EIIA^Glc^. These results suggest that regulation of ChiS by dephospho-EIIA^Glc^ is conserved across diverse strains of *V. cholerae* as well as in more distantly related *Vibrio* species.**

2. The manuscript presents a strong focus on EIIAGlc, and provides limited room for alternative hypotheses. Most data could also be explained by an indirect effect of EIIAGlc on ChiS (whether likely or not). I think the data need to be more carefully discussed in this view. To claim a direct interaction between ChiS and EIIAGlc, the hypothesis should be supported by biochemical work (crosslinking in Vibrio or in vitro, pulldowns using the two proteins, DNA binding assays in vitro when possible) showing a direct interaction [the POLAR assay can at best be interpreted as a likely interaction in a heterologous host and may depend on other proteins in such host]…In the absence of more concrete data, the claim of a direct interaction (e.g. L327) are insufficiently supported.

**The reviewer has suggested *in vitro* DNA-binding assays. While an excellent suggestion, biochemical assays with purified ChiS are unlikely to recapitulate the CCR activity observed *in vivo*. We are only able to purify the cytoplasmic fragment of ChiS. However, the primary suppressor we isolated in ChiS that confers resistance to EIIA^Glc^-mediated repression (W388C) is located within the protein’s HAMP domain, which extends through the membrane and into the periplasm. The conformation of the HAMP and the native association of ChiS with the membrane are, therefore, likely important for EIIA^Glc^-dependent repression, which would make this type of assay impractical to perform.**

**To bypass the problem posed by *in vitro* experiments with purified proteins, we performed the suggested experiment of crosslinking (using DSS, which has a spacer arm length of ~11.4 angstrom) in *V. cholerae* and performing *in vivo* pulldowns to test interactions between ChiS and EIIA^Glc^. To test whether ChiS interacts with EIIA^Glc^ in a phosphorylation-dependent manner, we performed pulldowns of ChiS-FLAG in strains where EI was intact vs deleted. We observed that when EI was intact, no EIIA^Glc^-mCherry could be detected in the elution fraction. However, when EI was deleted, a high molecular weight band was detected in the EIIA^Glc^-mCherry elution fraction, consistent with an interaction between EIIA^Glc^ and ChiS (Fig. 3D). Furthermore, introduction of the W388C suppressor mutation into the ChiS-FLAG construct prevented the coimmunoprecipitation of EIIA^Glc^-mCherry with ChiS (Fig. 5C), which is consistent with this suppressor mutation bypassing EIIA^Glc^-dependent repression of ChiS by preventing ChiS-EIIA^Glc^ interactions. Importantly, we confirmed that dephospho-EIIA^Glc^ represses ChiS activity in the same strains and conditions used for these pulldown assays using the P*_chb_*-*GFP* reporter (Fig. S5).**

**Our pulldown assay results strongly suggest a direct interaction between ChiS and EIIA^Glc^ - especially because of the relatively short spacer arm length in the crosslinker used. However, it is still formally possible that an intermediate protein is involved in this interaction. We therefore decided to further test this possibility. Specifically, we hypothesized that if an intermediate protein was required for EIIA^Glc^-dependent repression, that mutating this intermediate protein should relieve CCR of ChiS. To test this, we performed a mariner transposon mutant screen in a strain background that contained a P_chb_-*lacZ* reporter as well as ∆CBP (to activate ChiS) and ∆EI (to induce CCR of ChiS) mutations. This strain is typically white when plated on X-gal plates because the ∆EI mutation represses ChiS activity. We screened ~30,000 colonies in this screen, and isolated 5 candidate mutants where CCR of ChiS was relieved. All 5 of these mutants contained distinct Tn insertions in EIIA^Glc^, which suggests that the screen was saturating. However, no insertions were found in any genes other than EIIA^Glc^. These results suggest that no non-essential intermediate proteins are required for EIIA^Glc^-dependent CCR of ChiS activity.**

…also, if a specific interaction exists between EIIAGlc and ChiS, it should be possible to find suppressors in EIIAGlc as well to support this notion.

**The EIIA^Glc^ H91D allele is such an allele, albeit a targeted one. This mutant loses the ability to interact with ChiS. In other described EIIA^Glc^ targets (e.g., glycerol kinase), EIIA^Glc^ interacts with its targets via the area surrounding and including its phosphorylatable histidine (H91), which is what motivated us to test this allele in the first place.**

Modeling (e.g. Alphafold/Alphafold-Multimer) might also provide further support.

**We have performed Alphafold-Multimer modeling on ChiS and EIIA^Glc^ as suggested. Alphafold generates a quality score for the predicted interface (ipTM score). This score ranges from 0 to 1, with 1 being the highest quality/confidence. When a prediction is run with ChiS and EIIA^Glc^, the ipTM scores for the five models ranges from 0.22 to 0.14 (see Table 1 below). These are very low confidence values. However, this may not be surprising because Alphafold predictions are based, in part, on MSAs and evolutionary coupling. EIIA^Glc^ is known to have many targets, as discussed in this and other work, and its interactions with its targets are not obligate interactions. Thus, the coevolution between EIIA^Glc^ and its targets may not be robust enough for accurate prediction by Alphafold-multimer. In support of this hypothesis, we also tested a pair of proteins that are known to interact and for which a co-crystal structure has been solved = *E. coli* EIIA^Glc^ and glycerol kinase (PDB: 1GLA). Despite the fact that EIIA^Glc^ is known to interact with glycerol kinase, the ipTM scores for this pair were also low (ranging from 0.19-0.15, see Table 2 below). Thus, the Alphafold-multimer analysis has unfortunately not helped advance our understanding of the interaction between ChiS and dephospho-EIIA^Glc^.**

**Table 1: EIIA^Glc^ and ChiS**

| **Model Rank** | **ipTM** |
| --- | --- |
| 1 | 0.22 |
| 2 | 0.20 |
| 3 | 0.20 |
| 4 | 0.16 |
| 5 | 0.14 |

**EIIA^Glc^ and GlpK**

| **Model Rank** | **ipTM** |
| --- | --- |
| 1 | 0.19 |
| 2 | 0.16 |
| 3 | 0.16 |
| 4 | 0.15 |
| 5 | 0.15 |

Does the phosphorylation state of EIIAGlc affect the phosphorylation state of the other components (as one would expect in a relay) and if so, how does this factor into the results?

**The phosphorylation cascade starts with the phosphorylation of EI by PEP. This phosphate is transferred from EI to Hpr before being transferred to sugar-specific EIIA and EIIBC proteins. Finally, it is added to the incoming sugar molecule. The deletion of EI prevents phosphorylation of Hpr, EIIA^Glc^, and EIIBC^Glc^. If dephosphorylated Hpr were responsible for repression, the deletion of EIIA^Glc^ in the ∆EI background would have no effect on repression. Similarly, if EIIBC^Glc^ were involved in repression, an EI/EIIA^Glc^ deletion should phenocopy an EI deletion, which is not the case. The rescue of the ∆EI phenotype by the simultaneous deletion of EIIA^Glc^ specifically implicates dephosphorylated EIIA^Glc^ in the observed repression, and not other dephosphorylated members of the PTS system.**

1. Figures are not consistent: why are y-axis for the same variable (Fluorescence in AU, though beit mCherry and GFP in different panels) sometimes given on a linear scale and sometimes on a log scale? This complicates interpretation of the data.

**For cells grown in LB (for chitin-independent assays), fluorescence measurements were made using a plate reader, while for cells grown on chitin (for chitin-dependent assays), fluorescence measurements were made via microscopy. Microscopy provides a multi-log dynamic range, so displaying data on a log scale is more appropriate, and allows for better visualizing differences between groups. In contrast, the dynamic range of the plate reader-based assays is more limited (~1.5-log), so displaying data on a linear scale allows for better visualizing differences between groups.**

Similarly, why is data sometimes represented by single datapoints (e.g. Fig 1B) and sometimes in a bar chart?

**To make the figures more consistent, we changed our presentation of the single-cell data from Fig. 1B and similar figures. Rather than display the fluorescence emitted by single cells, we took the geometric mean from each replicate (~200 cells analyzed per replicate) and plotted those values.**

Some of the graphs (e.g. S2 are difficult to read and might benefit from additional color coding for specific groups. For traceability, I suggest including relevant strain names in the legend of Figures (see column Reference in manuscript column). Table S2: why do strains have double identifiers? Are these different strains? If they are the same, why list two identifiers?

**We have added color coding to several graphs, including Fig. S2. We have also added color to bars to help signify the sample that other bars are being compared to in order to simplify making comparisons.**

**The double identifiers in Table S2 refer to the same strain and are for organizational purposes. In our lab, each student generates an individual strain collection, which are named using their initials (e.g., VEG####). If strains are published, they are replicated and added to the permanent lab collection and are therefore given a separate SAD#### strain designation. We have clarified this as a footnote in the table to avoid confusion.**

**We believe that including the strain designations within the figures would be visually overwhelming and confusing. Instead, we have added additional clarifying text explaining the ‘Reference in Manuscript’ column.**

3. A key to incorporating the present findings in our thinking of chitin-inducible gene expression is the model as presented in Figure 7. Unfortunately, in its present form this figure is not very clear. I suggest concrete steps to improve the figure: a) a more schematic approach in which elements are aligned, less angled and where possible further simplified in shape; b) a more user-friendly color scheme (including only using color for those elements that are crucial to the model); c) a clear description of all elements in the Legend (at present for instance Glc and Glc~P are not described).

**The model figure was remade according to these excellent suggestions.**

Minor points:
2. Introduction: how relevant is CCR in the natural habitat of Vibrio. Are Glc levels sufficient for repression expected? I think it would be relevant to make a statement to this effect in the Introduction.

**We have amended the text in the Introduction to discuss the possibility of *V. cholerae* cells encountering transiently available EIIA^Glc^-dependent sugars released from decomposing wildlife. We further explore the potential relevance of CCR in the environment in the Discussion section, where we also discuss the possibility that the shutdown of chitin utilization and natural transformation by glucose is relevant to the transition of cells from the environment to the gut (as these expensive processes may not be useful in this context). We also discuss the possibility of an autoregulatory effect wherein the slow liberation of the GlcNAc monomer during chitin degradation begins to increase EIIA^Glc^ dephosphorylation and therefore begins to dampen chitin utilization. This effect would likely be subtle as the primary product of chitin breakdown is chitobiose rather than the GlcNAc monomer.**

3. Authors state that EIIAGlc is also responsible for GlcNAc import (L69) and that GlcNac is a constituent of chitin (L37). How does this fit into the authors statement of a “switch” between glucose and chitin utilization (L47-48)?

**The dominant product of chitinase activity is not the monosaccharide GlcNAc, but the disaccharide chitobiose. Aside from the possibility of a subtle autoregulatory effect after extensive digestion, GlcNAc production would be minimal compared to chitobiose production. This was included in the original Discussion, but we have added some text to make this point clearer.**

4. Fig 1B: what is the level of fluorescence in this assay for a strain lacking Pchb-mCherry (or ideally having a promoterless mCherry)? The authors claim “full repression by EI deletion”(L128) but levels are significantly above that of the chiS mutant. Please rephrase.

**The fluorescence microscopy data shown on these graphs has had background removed (fluorescence from a non-fluorescent strain has been subtracted). Therefore, all fluorescence observed represents a departure from background, or basal expression of the locus even in the absence of ChiS. The difference between ∆*chiS* and ∆EI is indeed statistically significant (see added statistics in Fig. 1B), suggesting incomplete transcriptional repression. We have clarified this in the text. Interestingly, this degree of transcriptional repression is sufficient to fully repress chitin utilization (Fig. 1C), but not natural transformation (Fig. 1D). However, repression is statistically significant for both behaviors and both transcriptional assays (Fig. 1).**

5. L140: what data in Fig 1B supports this claim?

**L139-142 states “Interestingly, the addition of cAMP did restore Pchb expression in the ∆EI/EIIAGlc strain back to parent levels of activity (Fig. 1B, Fig. S1B), suggesting that a reduction of cAMP in this background (due to low adenylate cyclase activity in the absence of phosphorylated EIIAGlc) does play a small role in limiting ChiS transcriptional activity.” This point is actually supported solely by data in Fig. S1B, which shows the addition of cAMP increases ChiS transcriptional activity. Thus, we have removed the reference to Fig. 1B from this statement.**

6. Figure 2: lacks a control of a chiS mutant (or a FLAG-less allele). This is a necessary control for the specificity of the ChIP data, as well as an interpretation of the enrichment still observed in the CBP/EI. Legend and Figure should indicate that these are data from a FLAG-tagged ChiS (the authors state it is fully functional, but data is NOT derived from a non-tagged construct).

**To address this point, ChIP assays were performed using an untagged ChiS allele. Enrichment in this sample was approximately 100-fold less than that observed in the ChiS-FLAG background where EI is intact. And approximately 2-fold less than the ChIP-signal observed in a ChiS-FLAG ∆EI background. These data suggest a very low level of ChiS-FLAG DNA-binding in the EI mutant, which can be detected by ChIP-qPCR. As a result, we have amended the text to avoid referring to repression as complete.**

**Previously published work has established that the ChiS-FLAG allele used here is fully functional (PMID: 32719134). This reference is provided in the text. We also show that the ChiS-FLAG allele is transcriptionally active and repressed by dephospho-EIIA^Glc^ in new data shown in Fig. S5.**

7. Fluorescence colocalization studies (Fig 3A and Fig 5A) are not very convincing – in particular, EIIAGlc foci are rather poor; perhaps this I the rendering of the figure and it can be improved by better pictures. But a higher resolution method or FRET/BRET-based approach would be preferential to demonstrate colocalization more conclusively. Based on this data, I do not agree with the statement that in L253.

**New images are provided, which we think more convincingly demonstrates the colocalization. Also, as discussed above, we now provide biochemical evidence that demonstrates that ChiS and EIIA^Glc^ interact, which is consistent with this colocalization data.**

Have all data underlying the figures and results presented in the manuscript been provided?
Large-scale datasets should be made available via a public repository as described in the PloS Genetics  [data availability policy](https://nam12.safelinks.protection.outlook.com/?url=http%3A%2F%2Fjournals.plos.org%2Fplosgenetics%2Fs%2Fdata-availability&data=05%7C01%7Cvegreen%40iu.edu%7C75bc6bc85da546d890b408dabcd6d3c7%7C1113be34aed14d00ab4bcdd02510be91%7C0%7C0%7C638029928118088381%7CUnknown%7CTWFpbGZsb3d8eyJWIjoiMC4wLjAwMDAiLCJQIjoiV2luMzIiLCJBTiI6Ik1haWwiLCJXVCI6Mn0%3D%7C3000%7C%7C%7C&sdata=HMT4SOBnBHHAMl%2Bup4JoPQ2HvsJ5WcRhpZoHxiC32s8%3D&reserved=0), and numerical data that underlies graphs or summary statistics should be provided in spreadsheet form as supporting information.

Reviewer #1: Yes

Reviewer #2: No**:** To my knowledge, the raw data underlying the graphs is not made available (see also point about data presentation)

**As suggested, we have added individual datapoints to all graphs and submitted a spreadsheet (S1 dataset) containing summary statistics for all data.**
